# Responsiveness to Sugar Solutions in the Moth *Agrotis ipsilon*: Parameters Affecting Proboscis Extension

**DOI:** 10.3389/fphys.2019.01423

**Published:** 2019-11-26

**Authors:** Camille Hostachy, Philippe Couzi, Melissa Hanafi-Portier, Guillaume Portemer, Alexandre Halleguen, Meena Murmu, Nina Deisig, Matthieu Dacher

**Affiliations:** Sorbonne Université, Université Paris Est Créteil, INRA, CNRS, IRD – Institute for Ecology and Environmental Sciences of Paris (iEES Paris), Paris, France

**Keywords:** moth, sugar responsiveness, dose-response curves, nectar, sugar, quinine, gustatory perception, proboscis extension response

## Abstract

Adult moths need energy and nutrients for reproducing and obtain them mainly by consuming flower nectar (a solution of sugars and other compounds). Gustatory perception gives them information on the plants they feed on. Feeding and food perception are integrated in the proboscis extension response, which occurs when their antennae touch a sugar solution. We took advantage of this reflex to explore moth sugar responsiveness depending on different parameters (i.e., sex, age, satiety, site of presentation, and composition of the solution). We observed that starvation but not age induced higher response rates to sucrose. Presentation of sucrose solutions in a randomized order confirmed that repeated sugar stimulations did not affect the response rate; however, animals were sometimes sensitized to water, indicating sucrose presentation might induce non-associative plasticity. Leg stimulation was much less efficient than antennal stimulation to elicit a response. Quinine prevented and terminated sucrose-elicited proboscis extension. Males but not females responded slightly more to sucrose than to fructose. Animals of either sex rarely reacted to glucose, but curiously, mixtures in which half sucrose or fructose were replaced by glucose elicited the same response rate than sucrose or fructose alone. Fructose synergized the response when mixed with sucrose in male but not female moths. This is consistent with the fact that nectars consumed by moths in nature are mixtures of these three sugars, which suggests an adaptation to nectar perception.

## Introduction

Moth reproduction and its regulation by sex pheromone have been largely studied to find ways to control their populations, as their larvae are important crop pests (Cook et al., 2007; Naranjo et al., 2015; Evenden, 2016). While less exploited for managing these pests, adult moths feeding behaviors and gustatory perception are also important for their reproduction; they need energy and nutrients to produce an abundant and healthy offspring (Hill, 1989; Hill and Pierce, 1989; Boggs and Ross, 1993; Wheeler, 1996; Boggs, 1997a,b; O’brien et al., 2000, 2004; Fischer et al., 2004; Jervis et al., 2005; Geister et al., 2008; Marchioro and Foerster, 2013; Levin et al., 2017a,b). Their food consists largely in sugars from flower nectars, which contains mainly fructose, glucose, and sucrose as well as a few amino acids and, sometimes, repellent substances (Baker and Baker, 1973; Baker, 1977; Lüttge, 1977; Gottsberger et al., 1984; Adler, 2000; O’brien et al., 2000, 2004; Pacini et al., 2003; De La Barrera and Nobel, 2004; Adler et al., 2006; Heil, 2011; Nepi et al., 2012; Atwater, 2013; Roy et al., 2017). Thus, gustatory perception is important for moths to assess food quality and discriminate what is edible from what is toxic (Glendinning, 2002; Adler and Irwin, 2005; Tiedeken et al., 2014).

When their legs, antennae, or proboscis contact a sugar solution of sufficient concentration, moths extend their proboscis and use it to imbibe the solution. This proboscis extension response (PER) first reflects the integration of gustatory perception and motivation for sugar and then allows feeding. Sucrose-elicited PER has been described and involved in associative learning in restrained insects including moths (Hartlieb, 1996; Fan et al., 1997; Hartlieb et al., 1999a,b; Fan and Hansson, 2001; Skiri et al., 2005; Jorgensen et al., 2007), butterflies (Kroutov et al., 1999), bees (Menzel, 1999, 2012; Page and Erber, 2002; Sandoz, 2011; Giurfa and Sandoz, 2012; Giurfa, 2015) and flies (Fresquet, 1999; Chabaud et al., 2006); similar feeding-related responses exist in ants (Guerrieri and d’Ettorre, 2010; Perez et al., 2013), crickets (Matsumoto et al., 2015), and bugs (Vinauger et al., 2013; Labrousse et al., 2017). PER has been used by Scheiner and her colleagues to assess responsiveness to sucrose in bees and flies (Scheiner et al., 2004a,b, 2013; Mujagic et al., 2010). These authors demonstrated that sucrose responsiveness is correlated to important parameters of honey bee biology such as division of labor, and foraging decisions are modulated by sugar gustatory perception (see for instance Nachev et al., 2013). Sugar-induced PER has been used in various moth species (Hill and Pierce, 1989; Winkler et al., 2005; Jørgensen et al., 2007; Zhang et al., 2010; Minoli et al., 2012) using different sucrose concentrations and animals in different physiological conditions; however, the impact of the physiological state on sugar responsiveness is hardly known in these insects.

Thus, we aimed for the first time at specifically looking at sugar responsiveness in relation to different physiological parameters (i.e., sex, age, and level of satiety) in the moth species *Agrotis ipsilon.* Testing systematically different concentrations and kinds of sugars composing nectar (i.e., sucrose, fructose, and glucose) further allowed us to find the optimal parameters for releasing the PER. Our work was inspired by extensive work on sucrose responsiveness in bees and flies (Scheiner et al., 2004a, 2013). We tested if sucrose responsiveness can also be induced using other sensory pathways beyond perception by the antennae (i.e., legs) and modulated by other gustatory stimuli (quinine). We also uncovered a non-associative plasticity, sensitization for water.

## Materials and Methods

### Animals

This study was performed on adult *A. ipsilon* (Lepidoptera, Noctuidae), which is a native species of France. Animals were reared in our laboratory in INRA, Versailles, France. Males and females were separated at the pupal stage and kept in different climatic chambers (22°C, 60–70% relative humidity) under an inverted photoperiod (16 h of light, starting at 18 h). Newly emerged adults (i.e., animals having just completed the imaginal molt) were collected every day (so that their post-emergence age is known) and grouped by 10 in breeding boxes (20 × 11.5 × 5 cm) with *ad libitum* access to either sucrose solution (fed animals) or tap water (starved animals). The sucrose solution was 12% weight/weight (13.6% weight/volume). We did not use more than 5-day starvation delays as the moths start to be weak or even die after starving for 6 days. Moths provided with sucrose gain weight, whereas moths provided only with water lose weights (unpublished observation), and we often observed that they readily drink the 12% solution in their breeding boxes but hardly react to water. Thus, it is reasonable to assume the feeding statuses of water- and sucrose-fed moths are not the same.

Experiments were conducted under dim red light and started at the middle of the scotophase, when moths are the most active (highest responsiveness of males to the sex pheromone, sex pheromone release by females), under a temperature of 22–24°C and 65% relative humidity. Unless otherwise mentioned, experiments were performed on day 5 post-emergence. Five-day-old *A. ipsilon* are standardly studied because males of this age are sexually mature and respond to the female sex pheromone (Gemeno and Haynes, 2000). In the morning of the experiments, animals were fixed in plastic tubes made from 1 ml pipette cones cut to fit a moth, so that only the head (with antennae and proboscis) protruded. Their position was further assured by inserting a small ball of absorbing paper or cotton behind them in the tube and fixing the top of their body to the tube using adhesive tape. Legs were blocked by the tape or inside the tube, unless otherwise mentioned (section “Proboscis Extension Response Induced by Leg Stimulation” reports the effect of leg stimulation). Once restrained, animals were left to acclimatize in the experimental room until the beginning of the experiment (in the afternoon).

### Sugar Responsiveness Assay and Solutions Used

We adapted Scheiner’s protocol for honey bees and flies (Scheiner et al., 2004a,b, 2013) to moths. Assessing sugar sensitivity consisted in presenting solutions of increasing sugar concentration; a 10-min interval between each presentation was used, except in section “Sensitization and Presentation Timing,” where a 1-min interval was also used. A solution presentation consisted in touching both antennae of the animal with a wooden toothpick imbibed with the sugar solution during 1–4 s; a response was recorded if the animal released a PER, i.e., extended its proboscis beyond its position at the onset of the stimulation (the initial position varies from one animal to the other, but the occurrence of the proboscis extension can be observed unambiguously). Animals were not fed, and great care was taken to avoid touching the proboscis with the toothpick by coming from behind the head, while the proboscis extends in front of it. We defined PER rate as the proportion of animals releasing a PER upon presentation of a sugar solution on the antennae.

The solution concentrations were 0 (i.e., deionized water), 0.1, 0.3, 1, 3, 10, and 30% (weight/weight in deionized water, e.g., 30% is 3 g of sucrose in 7 ml of deionized water). These sugar concentrations correspond to 0.1, 0.4, 1.1, 4.3, 11.1, and 42.9% weight/volume, respectively. A second presentation of the 0% solution was made afterwise to monitor the occurrence of sensitization, i.e., the fact animals might start to respond to weak stimuli such as 0% solution just because they are excited by previous sugar presentations. Sensitization is discussed in section “Sensitization and Presentation Timing.” This set of concentrations is inspired of previous studies in bees and flies (Scheiner et al., 2004a, 2013) and corresponds to a logarithmic increase in the stimulus intensity (i.e., around −1, −0.5, 0, 0.5, 1, 1.5), consistently with Weber’s law in sensory physiology (Waddington and Gottlieb, 1990; Akre and Johnsen, 2014; Shafir and Yehonatan, 2014). In our experiments, the sugar used was usually sucrose, but fructose, glucose, and various mixtures of these sugars were also tested (see section “Response to Various Sugars and Sugar Mixtures”). We focused on these three sugars (obtained from Sigma) as they are the main constituents of flower nectar, the main food of adult moths: typically 10–15% of the fresh mass for each sugar, possibly up to 30% for sucrose (Lüttge, 1977; Gottsberger et al., 1984; Pacini et al., 2003; De La Barrera and Nobel, 2004; Roy et al., 2017). In some experiment (section “The Effect of Quinine on Sucrose Responsiveness”), animals were also presented with 100 mM quinine (Sigma).

To rule out the possibility of having animals responding to water rather than sugar, animals responding to the initial water presentation were not kept in the analysis. However, removing these animals hardly modified the results. Supplementary data report figures including all the animals and the corresponding analysis (Supplementary Figures S1–S6, Supplementary Tables S13–S24).

### Experiments

#### Age, Sex, and Feeding Status

First, we investigated the effect of age and feeding status. Sucrose responsiveness was measured for various sucrose concentrations in five groups of males: 3-day-old moths either (1) with *ad libitum* access to sucrose solution (fed) or (2) starved (*ad libitum* access to water only), and 5-day-old moths either (3) fed, (4) starved for 3 days, or (5) starved for 5 days. Five-day-old moths starved for 3 days received sucrose from 0 to 2 days post-emergence and then only water for the following 3 days. Using this group was an attempt to discriminate between age of the animals and starvation duration, as when comparing 3-day-old and 5-day-old unfed moths, we simultaneously compare age (3-day-old or 5-day-old) and starvation duration (3 days or 5 days), so that the two factors are confused.

To assess the effect of sex, this experiment was replicated with females using the same age/starvation treatments, plus a group of 5-day-old male moths starved for 5 days for comparison purposes. Experiments reported elsewhere (Hostachy et al., submitted) indicated that sucrose responsiveness of males is not affected by the presence of sex pheromone, which allows to test them in parallel with females.

#### Sensitization and Inter-trial Interval

In a sucrose responsiveness assay, it is important to know whether responses (or lack thereof) to repeated presentations of sucrose of varying concentrations are independent from each other: having already been stimulated by a sucrose solution could affect responsiveness to the next simulations. This could be explained by sensitization (or habituation if we observed a decrease) and/or a time-dependent modification of motivation. To quantify this phenomenon, we performed the sucrose responsiveness assay in two groups of 5-day-old female moths: one group was presented with the sucrose solutions in the same order as previously (ascending concentrations), whereas for the other group, the six sucrose solutions were presented in a randomly determined order for each animals (keeping water as first and last stimulation). If sensitization or motivation variations alter sugar responsiveness when the solutions are presented in ascending order, then sugar responsiveness will be different in the random order group. Within each group, a sub-group was unfed for 5 days, whereas the other sub-group was fed; this was done to evaluate whether the occurrence of sensitization or motivation variations differed between fed and unfed animals.

To further explore the occurrence of sensitization and determine the importance of the inter-presentation interval, we used two inter-trial intervals: either 10 min (as previously) or 1 min. Indeed, a potential sensitization should occur more easily with a shorter inter-trial interval, as this is a form of short-term plasticity. In some animals, we also interleaved water presentations between sucrose presentations, as this was initially done in some experiments with bees to try and prevent sensitization (see for instance, Scheiner et al., 2003). For these groups, the interval between two sucrose presentations was either 2 or 20 min, with a water presentation interleaved at 1 or 10 min, respectively.

#### Effect of the Stimulation Site

While proboscis extension is classically elicited by antennal stimulation in bees and moths, it can also be triggered by leg stimulation (which is standard for fruit flies). Thus, we performed again the sucrose sensitivity protocol using two groups of animals, stimulating either the antennae (as previously, reference group) or the tibia and tarsi of the front legs. Leg-stimulated animals were restrained slightly differently: one of their front leg was fixed, so that it stayed outside of the tube. During leg stimulations, great care was taken to avoid touching the antennae. After testing all sucrose concentrations using the legs, the leg-stimulated animals were then assessed a second time using the antennae (i.e., the whole protocol was completed using the legs, then performed again using the antennae).

The proboscis also bears gustatory sensilla, so that stimulating it should elicit a PER. However, in moths, it is quite inconvenient to reach when it is coiled in its rest position; furthermore, it is impossible to prevent the animals to imbibe some of the solution in this case. Thus, evaluating sucrose responsiveness by direct proboscis stimulation is not feasible with this protocol.

#### Effects of a Bitter Compound: Quinine

As in nature moths consume sugar solutions found in flowers, they can be exposed to alkaloids that might have a deterrent effect. To assess whether alkaloids such as quinine affect PER and sucrose responsiveness, we performed again the sucrose responsiveness protocol on 5-day-old males. Animals releasing a PER had their proboscis immediately touched either with water (control group) or with 100 mM quinine, and we recorded whether the PER would continue after this proboscis stimulation. To complement these observations, we also measured responsiveness to sucrose and sucrose plus 100 mM quinine in 5-day-old males.

#### Effects of Other Sugars: Fructose, Glucose, and Sugar Mixtures

Sucrose is the reference sugar used in PER experiments in various insects. However, it is well known that nectar can also include fructose and glucose in various proportions. Thus, we measured responsiveness for fructose and glucose besides sucrose, as well as mixtures between these three molecules. For mixtures, the same concentrations were used with sugars in equal amounts (i.e., a 3% sucrose/fructose solution contains 1.5% sucrose, 1.5% fructose, weight/weight; or 2.15% of each sugar, weight/volume). Experiments were done with 5-day-old males unfed for 5 days.

In another experiment, the sugar responsiveness assay was performed in females with glucose, fructose, and sucrose, as well as sucrose/fructose and sucrose/fructose/glucose mixtures. All animals were 5-day-old females unfed for 5 days, and a group of 5-day-old males unfed for 5 days and tested with sucrose was also used for comparison purpose.

### Data Analysis

Data were analyzed using R 3.4.0 (R Core Team, 2017) and RStudio 1.1.423, taking an α risk of 0.050. PER rates were computed for each experimental group and are reported in the figures, with the sample sizes reported in parenthesis in the legends. Thus, a dose-response curve was obtained for each experimental group.

For each sugar concentration in a dose-response curve, PER rates were compared between experimental groups using *χ*^2^ test or bilateral Fisher’s exact test when *χ*^2^’s assumption (Cochran’s rule) was not met. When more than two groups were compared and a significant *p* was obtained, we performed pairwise comparisons using the same tests and adjusted *p* for repeated testing using Holm’s method (Holm, 1979); however, it sometimes happened adjusted *p* could not determine the source of the difference detected in the global test.

In a few cases, response variations within a group were compared using McNemar’s test, using Holm’s method to adjust *p* when needed. Logistic regression (binomial generalized linear model) was also used in section “Sensitization and Presentation Timing” to assess whether PER rates to the final water presentation were affected by the sugar concentration presented 10 min before or by the number of PER previously released (preliminary stepwise analysis indicating the interaction term was not significant, so that it was dropped). To avoid tedious lists of *p* in the results, most of them are only presented in tables in supplementary data (Supplementary Tables S1–S12, one table for each figure or figure panel).

## Results

### The Effect of Age, Starvation Duration, and Sex on Sucrose Responsiveness

**Figure 1:**
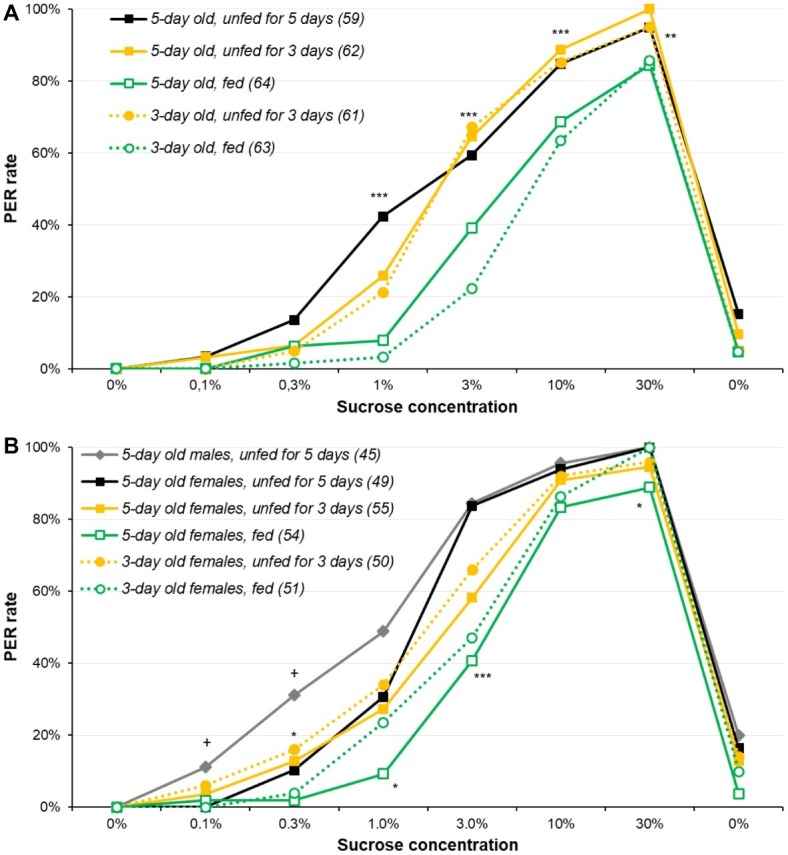
Effect of age and starvation duration on sucrose responsiveness in *Agrotis ipsilon*. The plots present dose-response curves for various sucrose concentrations (*x*-axis); the *y*-axis reports the PER rate, i.e., the proportion of insects extending their proboscis when their antennae were briefly touched with a drop of sucrose solution. Each sucrose solution presentation was separated by 10 min. In the legend, numbers in parenthesis correspond to the sample sizes. In part **(A)**, experiments were done with males; in part **(B)**, experiments were done with females and a group of males for comparison purpose. Stars denote significant differences between male or female groups in *χ*^2^ or Fisher’s exact tests (^*^p < 0.050; ^**^p < 0.010; ^***^p < 0.001); in part **(B)**, crosses denote significant differences between 5-day old males and females unfed for 5 days in *χ*^2^ of Fisher’s exact test (^+^*p* ≤ 0.050). Details of the analyses are reported in Supplementary Table S1 for part **(A)** and Supplementary Table S2 for part **(B)**.

We first explored the experimental parameters required for optimally assessing sucrose responsiveness by comparing age and starvation duration. For male moths (Figure 1A and Supplementary Figure S1A), significant differences were observed for all sucrose solutions of 1% and higher (*χ*^2^ or Fisher’s exact test, *p* ≤ 0.004): overall, fed animals were less responsive than unfed ones, irrespective of their age, while 5-day-old moths unfed for 5 days were consistently among the highest PER rates (details of the pairwise comparisons are in Supplementary Table S1).

For females (Figure 1B and Supplementary Figure S1B), results were analyzed in two steps: first, 5-day-old males and females unfed for 5 days were compared, and second, all female groups were compared. For the lowest concentrations (0.1 and 0.3%), the PER rates of females were slightly lower than males’ (*χ*^2^ or Fisher’s exact test, *p* ≤ 0.022), suggesting females are slightly less responsive than males for the lowest concentrations. However, the same pattern as for males was observed for the PER rates across treatments, i.e., 5-day-old females unfed for 5 days were consistently among the highest PER rates for 0.3, 1, 3, and 30% concentrations (details of the pairwise comparisons are in Supplementary Table S2).

### Sensitization and Presentation Timing

**Figure 2:**
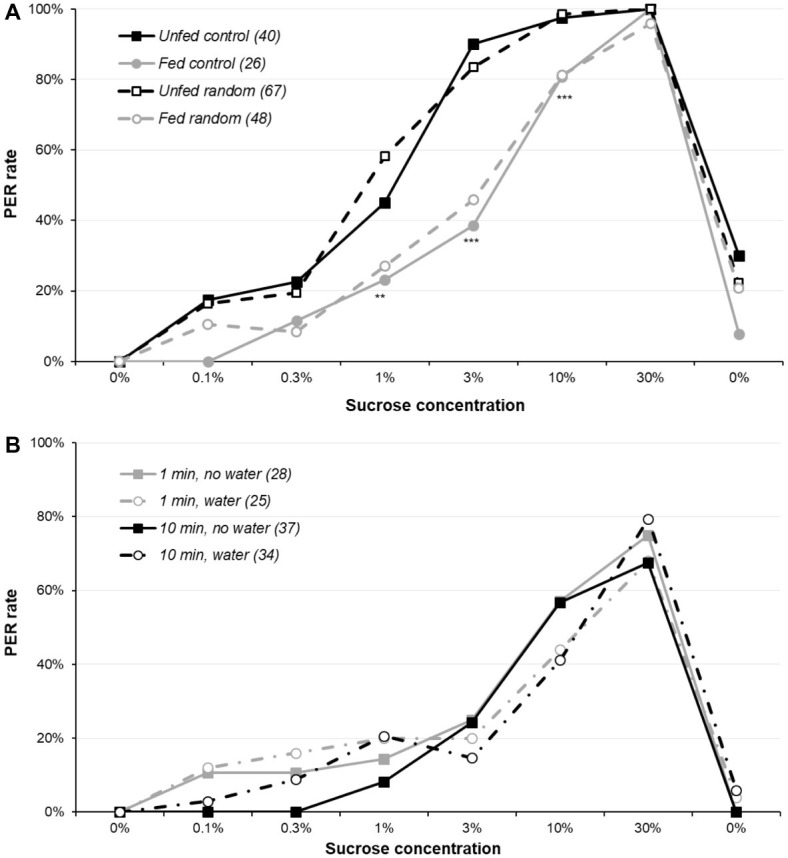
Sensitization and sucrose responsiveness. Part **(A)** reports sucrose responsiveness in four groups of 5-day-old females: the first two were presented sucrose concentrations in ascending order (as previously), whereas for the other two, the six sucrose solutions were presented in an order randomly determined for each animal. Within each treatment, one of the two groups had been unfed for 5 days, whereas the other was fed. Part **(B)** plots the sucrose responsiveness in four groups of unfed 5-day-old males: two groups with an inter-trial interval of 10 min (as previously) and two groups with an inter-trial interval of 1 min. For each interval, one of the two groups had a water presentation every other trials, so that for these groups, the interval between two sucrose presentations was either 2 or 20 min, with a water presentation interleaved at 1 or 10 min. Some animals in the two no-water groups were not presented the final water solution. Other details are as in Figure 1. Details of the analyses are reported in Supplementary Table S3 for part **(A)** and Supplementary Table S4 for part **(B)**.

When the independence of the stimulations and the occurrence of sensitization was assessed (Figure 2A and Supplementary Figure S2A), there was a global significant difference between the four groups for 1, 3, and 10% sucrose concentrations (*χ*^2^ or Fisher’s exact test, *p* ≤ 0.001), replicating the previous finding that fed animals have a lower sucrose responsiveness; by contrast, presenting the solutions in random order or in ascending order did not have any effect (Supplementary Table S3 reports detailed analysis). As a result, sensitization (or even habituation) or variations of motivations during the assay are unlikely to be an important determinant of the PER rate in the sugar responsiveness assay.

However, even if sensitization does not alter sucrose responsiveness, it could explain the PER rate observed during the final water presentation. To test this, we used the data from the unfed random group and computed a logistic regression to test the effect of two factors on water responsiveness: the total number of responses before the final water test (ranging from 0 to 6, i.e., the number of responses to the 6 sugar concentrations) and the concentration of the sugar solutions used before water (which can be any of the 6 concentrations in this random order group). Both factors were significant (logistic regression *p* ≤ 0.046). This indicates that animals responding to most sucrose concentrations tend to respond to the final water, even though they did not respond to the initial water presentation. Moreover, a higher concentration just before the final water presentation (typically, the 3, 10, or 30% solutions) also contributes to this sensitization to water.

Figure 2B and Supplementary Figure S2B report sucrose responsiveness with various inter-trial intervals and with or without interleaved water. Our hypothesis was that sensitization would occur in the 1-min group without interleaved water and be lower in the 10-min group with interleaved water, but this was not the case: the four groups were similarly responsive (*χ*^2^ or Fisher’s exact test, *p* ≥ 0.064; details of the analysis are in Supplementary Table S4). Moreover, responses to the interleaved water presentation were low and did not differ significantly across groups (data not shown; Fisher’s exact test, *p* ≥ 0.386).

### Proboscis Extension Response Induced by Leg Stimulation

**Figure 3:**
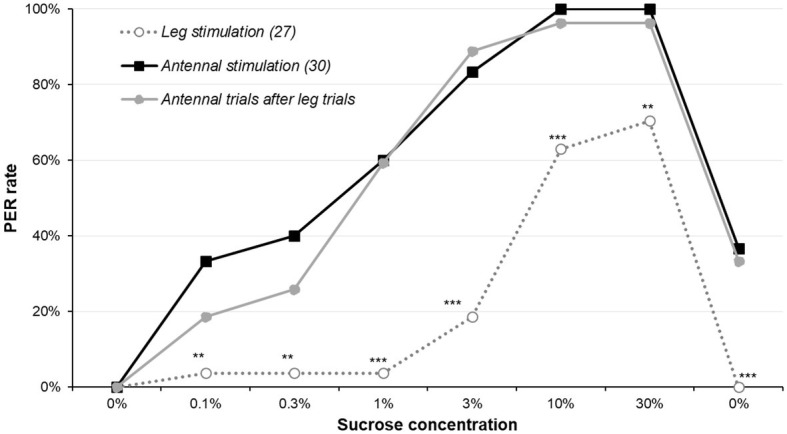
Comparison of antennal stimulation and leg stimulation. The sucrose sensitivity experiment was conducted by stimulating either the antennae (as previously) or the legs of 5-day-old males starved for 5 days. Animal stimulated on the legs for the complete set of sucrose solutions underwent the protocol a second time with the antennae (i.e., the whole set of sucrose solutions was presented twice, one on the legs and the other on the antennae). Stars denote significant difference between the leg stimulated group and the antennal stimulated group run in parallel (*χ*^2^ or Fisher’s exact test; ^**^p < 0.010; ^***^p < 0.001). Details of the analyses are reported in Supplementary Table S5. Other details are as in Figure 1.

Results on the effect of leg stimulation are presented in Figure 3 and Supplementary Figure S3 and details of the statistics in Supplementary Table S5. Leg stimulation was much less efficient to elicit PER, as it only did so to a meaningful level for the highest concentrations; in all cases, leg-elicited-PER rate was significantly smaller from antennal-elicited-PER rate (*χ*^2^ or Fisher’s exact test, *p* ≤ 0.005). A second series of tests was made to compare the leg group to the reference group when the leg group was retested on the antennae: this time, there was no difference between the two groups (*χ*^2^ or Fisher’s exact test, *p* ≥ 0.205). This means that the leg-stimulated group had the same sucrose responsiveness as the control when tested on the antennae. This confirms that the lower PER rate is only due to the different types of stimulation.

### The Effect of Quinine on Sucrose Responsiveness

**Figure 4:**
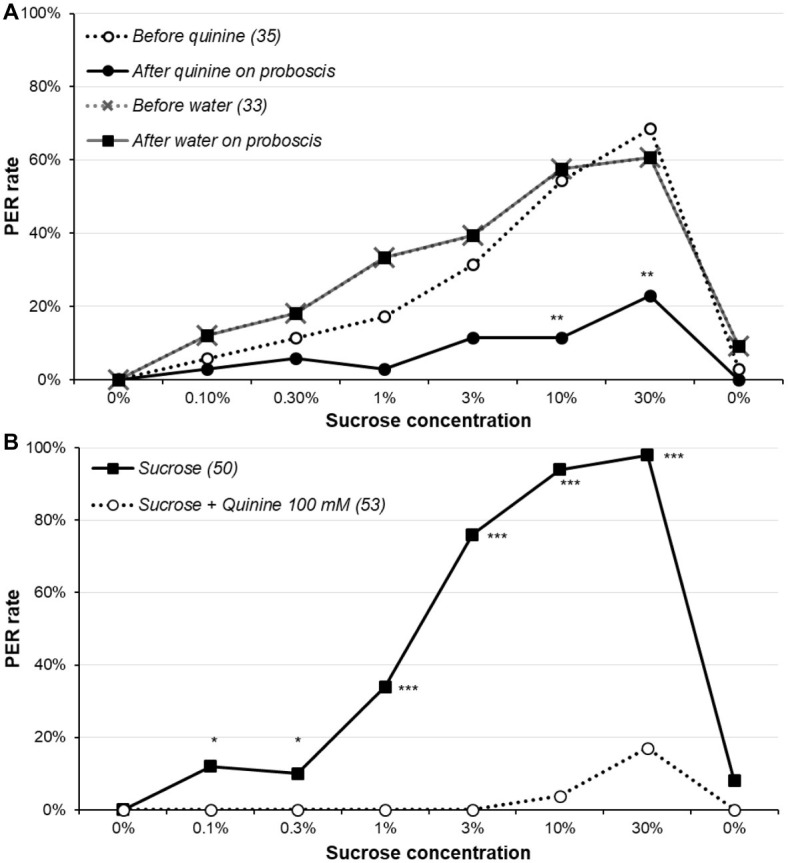
Effect of quinine on sucrose responsiveness. In part **(A)**, animals were presented the series of sucrose solutions (dotted lines), and if they released a PER, they immediately received either 100 mM quinine or water on the extended proboscis; the continuous lines indicate the proportion of animals still releasing a PER after receiving one of these solution. Notice that as touching the proboscis with water had no effect, the two lines (before and after water) are superimposed. Stars denote a significant difference between the PER rate before and after touching the proboscis with quinine, i.e., a significant rate of proboscis retraction (McNemar’s test ^**^p < 0.010); detailed analysis is in Supplementary Table S6. In part **(B)**, animals were presented the series of sucrose solutions either alone as previously or combined with 100 mM quinine. Stars denote a significant difference between the PER rate of the two groups (*χ*^2^; ^*^p < 0.050, ^***^p < 0.001); detailed analysis is in Supplementary Table S7. All animals were 5-day-old males starved for 5 days; other details are as in Figure 1.

The effects of quinine stimulation are reported in Figure 4A and Supplementary Figure S4A and the detailed analysis in Supplementary Table S6. Before contacting the proboscis with water or quinine, the sucrose responsiveness did not significantly differ between the water- and the quinine-stimulated group (*χ*^2^ or Fisher’s exact test, *p* ≥ 0.123), confirming the two groups were identical at this step. Touching the proboscis with water did not affect the PER, as no animal retracted its proboscis; by contrast, most animals which proboscis touched quinine interrupted the PER and retracted the proboscis, almost significantly for sucrose concentrations 1 and 3% (McNemar’s test, *p* ≤ 0.071), and significantly for concentrations 10 and 30% (McNemar’s test, *p* ≤ 0.005); no difference was observed for other concentrations, but the PER rates were low for them anyway. We also compared the proportions of animals retracting the proboscis among those initially responding, and the results were the same (data not shown). Interestingly, the fact that the PER rates remain the same as in the control group in ulterior sucrose solution presentations means that even though quinine elicited proboscis retraction, it does not modulate sucrose responsiveness (at least after 10 min). Moreover, when quinine was added to sucrose (Figure 4B, Supplementary Figure S4B, Supplementary Table S7), it strongly inhibited PER (*χ*^2^ or Fisher’s exact test; *p* ≤ 0.024).

### Response to Various Sugars and Sugar Mixtures

**Figure 5:**
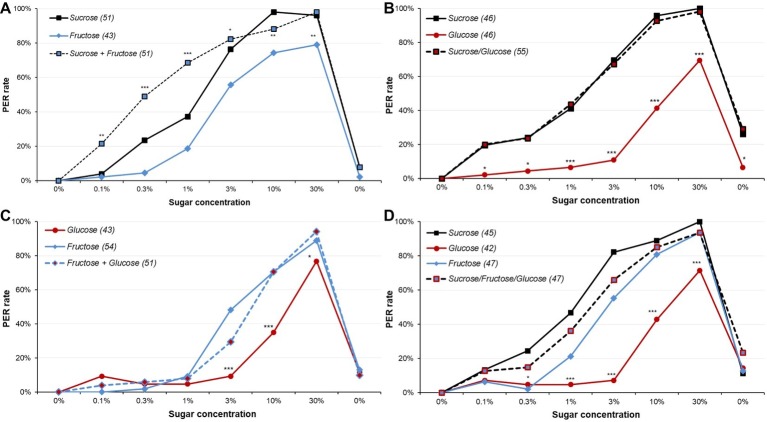
Responsiveness to various sugars and sugar mixtures in male moths. These plots display dose-response curves for various sugars and sugar mixtures. Part **(A)** presents PER rates for fructose, sucrose, and their mixture; part **(B)** for glucose, sucrose, and their mixtures; part **(C)** for glucose, fructose, and their mixture; and part **(D)** for glucose, fructose, sucrose, and their mixtures. All mixtures involved even amount of sugars. All animals were 5-day-old male moths starved for 5 days. Other details as in Figure 1. Detailed analysis is reported in Supplementary Tables S8–S11.

In a first experiment, animals were presented with either sucrose, fructose, or a mixture of these sugars (Figure 5A and Supplementary Figure S5A, Supplementary Table S8). Although fructose elicited high PER rates, overall they were significantly lower than for sucrose (*χ*^2^ or Fisher’s exact test; adjusted *p* = 0.068 for 3%, *p* ≤ 0.046 for 0.3, 1, 10, and 30%). Interestingly, mixtures of fructose and sucrose elicited the same level of PER rate as sucrose alone (*χ*^2^ or Fisher’s exact test; adjusted *p* ≥ 0.217 for 3, 10, or 30%), or even higher responses for lower concentrations (*χ*^2^ or Fisher’s exact test; adjusted *p* ≤ 0.030 for 0.1, 0.3, or 1%). Thus, while fructose tended to elicit less responses than sucrose, replacing half the sucrose by fructose in a mixture actually improved the PER rate, indicating a synergistic effect.

In a second experiment, we compared animals’ responses to sucrose, glucose, and their mixture (Figure 5B and Supplementary Figure S5B, Supplementary Table S9). Glucose elicited PER much less often than the two other solutions (*χ*^2^ or Fisher’s exact test, adjusted *p* ≤ 0.020) and induced less sensitization for the final water presentation (*χ*^2^, adjusted *p* ≤ 0.022). Yet, replacing half the sucrose by glucose did not modify the PER rate relatively to the sucrose group (*χ*^2^ or Fisher’s exact test, adjusted *p* ≥ 0.686); thus, in spite the mixture only includes half sucrose found in the sucrose solution (e.g., the 3% mixture only includes 1.5% sucrose), and so should elicit much lower PER rates, the response is unchanged. This leads to the intriguing conclusion that while glucose did not elicit much response, replacing half of the sucrose by glucose in a solution did not decrease the response, although there was not the synergy observed for sucrose and fructose.

When the responses to fructose, glucose, and their mixture were compared (Figure 5C and Supplementary Figure S5C, Supplementary Table S10), results were similar: glucose elicited less response than fructose or fructose/glucose mixtures for 3 and 10% (*χ*^2^, adjusted *p* ≤ 0.031); for 30% sugar solution, the global test was significant (*χ*^2^, *p* = 0.038), but pairwise comparisons were not. Fructose and fructose/glucose mixtures elicited similar PER rates, except for the 3% concentrations, where the mixture was slightly inferior (*χ*^2^, adjusted *p* = 0.049). To sum up, while animals had lower PER rates for glucose than for fructose, replacing half of the fructose by glucose impaired only slight response. This is comparable to what was seen with sucrose/glucose mixtures.

Finally, we compared PER rates to sucrose, glucose, fructose, and a sucrose/glucose/fructose mixture (Figure 5D and Supplementary Figure S5D, Supplementary Table S11). We replicated the observation that fructose was slightly but significantly less responded to than sucrose (*χ*^2^ or Fisher’s exact test, adjusted *p* ≤ 0.040 for 0.3, 1 and 3%), and that glucose was much less responded to than fructose or sucrose for 1, 3, 10, and 30% (*χ*^2^ or Fisher’s exact test: adjusted *p* = 0.068 for 1% for glucose vs. fructose, adjusted *p* ≤ 0.046 for other comparisons). Moreover, glucose also elicited less PER than the mixture for 1, 3, 10, and 30% (*χ*^2^ or Fisher’s exact test, adjusted *p* ≤ 0.045), whereas sucrose or fructose did not differ from it (*χ*^2^ or Fisher’s exact test, adjusted *p* ≥ 0.151). Therefore, neither synergy nor inhibition was observed in this case.

**Figure 6:**
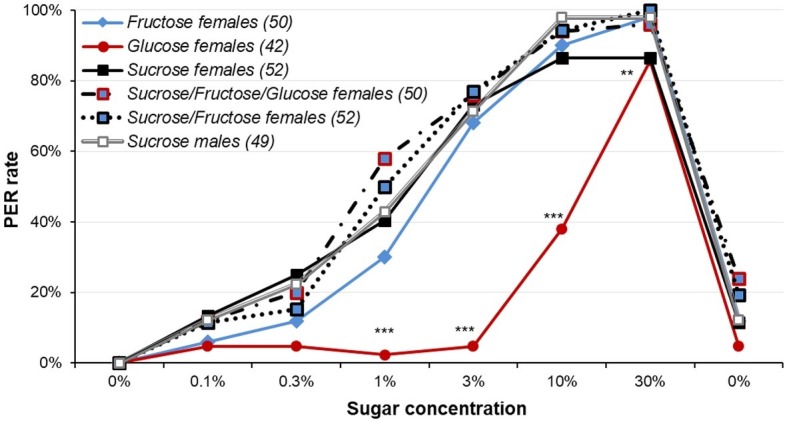
Responsiveness to various sugars and sugar mixtures in female moths. This plot reports dose-response curves for various sugar and sugar mixtures in 5-day old female moths starved for 5 days; a group of 5-day old males starved for 5 days was also used for comparison purpose. Sucrose, fructose, and glucose were used, as well as sucrose/fructose/glucose and sucrose/fructose mixtures. Stars denote significant differences between female groups (*χ*^2^ or Fisher’s exact test; ^**^p < 0.010; ^***^p < 0.001). Other details as in Figure 1. Detailed analysis is reported in Supplementary Table S12.

When females were tested for sugar mixtures (Figure 6 and Supplementary Figure S6, Supplementary Table S12), contrasting for what was seen in Figure 1B, males and females tested for sucrose did not differ for low concentration of sucrose (*χ*^2^, *p* ≥ 0.763) and males were only slightly higher than females for 10 and 30% sucrose, but without reaching significance (Fisher’s exact test, *p* = 0.060 in both cases). While there was no difference between the female groups for 0.1 or 0.3% sugar concentrations (*χ*^2^ or Fisher’s exact test, *p* ≥ 0.080 in both cases), there were significant differences for 1, 3, and 10% sugar concentrations (*χ*^2^ or Fisher’s exact test, *p* ≤ 0.004 in all cases). This difference was mainly due to much lower PER rates to glucose solution (*χ*^2^ or Fisher’s exact test, adjusted *p* ≤ 0.042 in all cases). The other groups did not differ (*χ*^2^ or Fisher’s exact test, adjusted *p* ≥ 0.113), except Fructose and Sucrose/Fructose/Glucose mixture for 1% (*χ*^2^, *p* = 0.029).

## Discussion

### The Sugar Responsiveness Assay: Impact of Various Parameters

This study assessed sucrose responsiveness in relation to different physiological states in the moth *A. ipsilon*. Sucrose responsiveness has been extensively studied in the honey bee (Scheiner et al., 2004a, 2013) and in some moths (Winkler et al., 2005; Zhang et al., 2010; Minoli et al., 2012). We used a broad range of concentrations, consistent with what was used in bees (Scheiner et al., 2004a, 2013) and flies (Scheiner et al., 2004b), and including concentrations found in nectar (10–30% for sucrose: Lüttge, 1977; Gottsberger et al., 1984; Pacini et al., 2003; De La Barrera and Nobel, 2004; Roy et al., 2017).

Our exploration of the parameters associated to sucrose responsiveness has led to various conclusions. Overall, sucrose responsiveness depends upon starvation duration (i.e., motivation for food) and possibly sex but not age; as a result, in most experiments, we used 5-day-old moths starved for 5 days as the standard condition. The inter-trial interval or the interleaving of water are not crucial parameters in the sucrose responsiveness assay, as the response of the animals is not influenced by the previous trials; yet, we could observe some sensitization to water, a new form of behavioral plasticity in *A. ipsilon* (see also Minoli et al., 2012 for a study of sensitization in a related species). Finally, while moths can detect sucrose through their legs, this pathway is less prone to elicit a PER, either because it does not respond to sucrose very well or because it is less well coupled with the PER motor pathways.

Beyond physiological parameters, the composition of the solution is also essential to observe the PER. In the presence of quinine, moths do not release PER. Thus, quinine is more aversive than sucrose is appetitive, i.e., its ability to elicit a withdrawal of the proboscis is stronger than sucrose’s ability to elicit its extension. A simple overshadowing of sucrose is unlikely, as it would not explain PER termination in Figure 4A. Concerning the sugars found in nectars, sucrose elicited more responses than fructose, and responses to glucose were much lower than either of them. However, replacing half of the sucrose or half of the fructose by glucose did not lower the PER rate, and mixing sucrose and fructose increased it, suggesting a synergy; interestingly, glucose did not support a synergistic effect in sucrose/fructose/glucose mixture. The results with females are quite different from those observed in males: sucrose and fructose are responded to at the same level, and mixing them (with or without glucose) had no effect. The common point is that glucose alone does not elicit much response while being able to replace the other sugars in mixture.

While simple, the sugar responsiveness assay offers many experimental opportunities. First and foremost, the words “sugar responsiveness” cover various underlying processes, which is why they were preferred to “sugar sensitivity.” The dose-response curves used here simultaneously assess three functions: perception (the ability to detect sugar), motivation for sugar (the ability of sugar perception to trigger a PER), and motor response (i.e., if a PER is triggered, the ability to produce it). Detecting sugar and releasing a PER are well-defined phenomena. By contrast, while motivation is associated to them (e.g., Figures 1 and 2 link hunger to PER rate), its definition is blurrier as it encompasses various situations: not only hunger (i.e., physiological need for food induced by starvation) but also the lack of satiety (i.e., animals’ desire to eat). While certainly correlated, these two situations are not necessarily the same; it would be possible to dissociate them by feeding animals with sweeteners, which induce satiety (i.e., they are palatable) but are physiologically non-nutritive (Schiff et al., 1989; Pszczolkowski and Brown, 2003; Dus et al., 2011; Camacho et al., 2017; Choi et al., 2017; Fisher et al., 2017; Takada et al., 2017; Mustard et al., 2018). The sucrose responsiveness assay would allow to test whether such a dissociation occurs in moth. Similarly, fed animals might have lower response not because they are hungry, but rather because they are habituated to sucrose; to test this hypothesis, animals could be reared (as adult or even as larvae) with fructose rather than sucrose, so that they would not be starved, but still naïve for sucrose. Another possibility offered by the sugar responsiveness assay is to assess lateralization of the response by presenting sugar on a single antennae rather than both; studying lateralization in invertebrate is an emerging field (Jozet-Alves et al., 2012; Baracchi et al., 2018; Niven and Frasnelli, 2018), which complements the classical studies of its role in primate higher cognitive abilities (Prieur et al., 2019). In moths, a study in *Spodoptera littoralis* reported sensitization elicited by pre-exposure to sucrose is a lateralized process (Minoli et al., 2012). All these possible experiments highlight the interest of the sucrose responsiveness assay to investigate precise questions on gustatory responses in moths. Understanding the functioning of sugar-elicited PER could help discriminating between perception, motivation, and PER release mechanisms.

Sensitization is a form of non-associative learning that would make an animal respond to a stimulus (here, the sucrose solution or water) just because it has been excited by a previous presentation of the same or another stimulus (Thompson and Spencer, 1966; Groves and Thompson, 1970; Hammer et al., 1994; Anderson et al., 2007; Anton et al., 2011; Minoli et al., 2012; Blumstein, 2016). Here, we carefully monitored this possibility by using a 10-min interval between each sugar presentation to avoid sensitization, which was hardly considered before in moth sucrose responsiveness. We found sensitization does not affect sucrose responsiveness, although it increases water responsiveness. Interestingly, it was shown in bees and flies that while a forward pairing (presentation of odor, then sugar) promotes associative learning, a backward pairing (sugar than odor) prevents it. This effect seems to be mediated by a desensitization of the PER occurring upon odor presentation at a specific 15-s delay (Dacher and Smith, 2008). Whether this type of desensitization also occurs in moths remains to be determined.

### Biological Meaning of Sucrose Responsiveness: Sugar Mixture, Foraging, and Nectar Composition

It is well established in bees that associations between odors and PER/sugar made in restrained conditions can be transferred to free-flying foraging, and vice versa (Gerber et al., 1996; Sandoz et al., 2000; Chaffiol et al., 2005; Gil and De Marco, 2005). Therefore, it is sensible to assume such transfers are possible between the two situations for moths too. Under this hypothesis, sucrose responsiveness is likely to play an important role in modulating foraging behaviors in these insects, as in bees; indeed, it is well established that bees foraging for nectar are less responsive to sucrose than bees foraging for pollen (Scheiner et al., 2004a). As a result, only the most concentrated nectar sources are exploited by bee nectar foragers. Such a phenomenon could also exist in most foraging moths, which land on flowers (i.e., settling moths but not hovering moths: Oliveira et al., 2004; Makholela and Manning, 2006; Okamoto et al., 2008; Atwater, 2013): it is likely the first appendage assessing sugar concentration in nectar are the legs (or possibly the proboscis) rather than the antennae (see also experiments by Zhang et al., 2010). Thus, only highly concentrated nectar would elicit a PER in moth (Figure 3), just like nectar-foraging bees. To explore this hypothesis, the foraging behavior of male and female moths drinking nectar at flowers should be analyzed either in artificial flowers in wind tunnel or in a natural setting. Very little information is available on this topic.

While moths respond quite well to sucrose and fructose, they hardly react to glucose. This is consistent to what was observed in a related species, *Spodoptera littoralis*, during electrophysiological recordings of taste sensilla (Popescu et al., 2013). In spite it is hardly responded to, glucose can replace fructose or sucrose in mixtures without significantly decreasing the PER rate. As nectars are mixtures of sugars rather than single compounded, this result suggests an adaptation to nectar perception. We can make the hypothesis that moths or other animals feeding on only one type of plant (i.e., strictly monophagous) should have a sugar responsiveness specifically tuned to the specific sugar ratio found in this plant’s nectar, illustrating plant-pollinator coevolution (e.g., Perret et al., 2001; Nicolson and Fleming, 2003; Lotz and Schondube, 2006). This would have important consequences for the management of pests and pollinators. This hypothesis is also consistent with the fact that learning performance depends on sugar identity in bees (Simcock et al., 2018; Chapuy et al., 2019). By contrast, polyphagous moths such as *A. ipsilon* feed on various types of flowers, possibly with differing nectars (Wynne et al., 1991; Zhu et al., 1993). Nectars can be classified in various broad categories (Percival, 1961), and usually glucose is neither the dominant sugar nor the only one. Thus, it makes sense moths detect it less well. Honey bees are much better at detecting it (Simcock et al., 2018), but it is one of the main constituent of their honey. It would be interesting to compare sucrose responsiveness of other nectar foragers, particularly pollinators.

Honey bees and *Agrotis ipsilon* are sympatric, and both are described as polyphagous (i.e., they are not specialized in a narrow range of plant species but rather forage nectar on various flowers; Wynne et al., 1991; Zhu et al., 1993); thus, they potentially forage on the same plants. However, previous results (Scheiner et al., 2004a, 2013) indicate bees are much more sensitive to low sucrose concentrations than moths and so are likely to explore a wider range of flowers, including those which have nectar less concentrated. A full exploration of the ecological and evolutionary implications of this preliminary observation is beyond the scope of this article, but it certainly deserves to be studied; indeed, nectar foraging insects are often pollinators, and it is relevant to know which plants will or will not be visited by a given insect in a context of pollination crisis (Senapathi et al., 2015). The sucrose responsiveness assay will be useful to do so.

We found little differences between males and females, and they were not consistently observed. It would be interesting to compare sugar responsiveness in animals before and after reproduction; for males, that would mean before flying toward a female and mating, and for females, before releasing pheromone, mating and laying eggs. It is not unlikely the needs of moths change according to their reproductive status. In particular, females might need amino acids, while eggs are storing reserves (Watt et al., 1974; Heil, 2011; Hendriksma et al., 2014; Simcock et al., 2014; Levin et al., 2017a,b), so that PER rates would be affected by their presence in the tested solutions (but see O’brien et al., 2000; Marchioro and Foerster, 2013 for contradictory results); Zhang et al. (2010) reported neuronal responses and PER to amino acids. Similarly, males might need sodium and potassium for spermatophore formation (Arms et al., 1974; Adler and Pearson, 1982; Boggs and Jackson, 1991; Smedley and Eisner, 1995, 1996; Beck et al., 1999; Boggs and Dau, 2004; Watanabe and Kamikubo, 2005; Molleman, 2010), so that the presence of salt in the solution might affect the PER rate. The sugar responsiveness assay can easily be modified to test the effect of salt or amino acid on males and females of different reproductive status.

Presenting quinine on the proboscis interrupts the PER, and it inhibits response to sugar. This contrasts to the behavior of bees (which readily drink bitter solutions when they are restrained but not when they are free flying, Ayestaran et al., 2010; De Brito Sanchez et al., 2015; Guiraud et al., 2018) and flies (which accepts bitter compounds after starvation, Ledue et al., 2016). It is established that some plants produce bitter compounds in nectar (Adler, 2000; Adler et al., 2006; Heil, 2011; Roy et al., 2017), possibly to deter predatory insects. It can be expected that *A. ipsilon* would not forage on these plants, but this remains to be verified; this would be consistent with the idea that this moth is a generalist forager, because specialists tend to adapt to deterrent nectar compounds (Berenbaum et al., 1989; Glendinning, 2002; Cornell and Hawkins, 2003; Adler et al., 2006; Reisenman and Riffell, 2015). This also opens the possibility to use the sugar responsiveness assay to evaluate moths’ reaction to aversive compounds such as deterrent products released in nectar, or possibly xenobiotics. Beside, some results indicate appetitive and deterrent stimuli are processed in parallel at the antenna level (Kvello et al., 2010); this raises the question of the neurophysiological convergence of these pathways, which must occur at some point as quinine prevents sucrose-induced PER. Interestingly, pre-exposing *S. littoralis* to quinine or sucrose 24 h before sucrose presentation potentiates rather than inhibits the response to 1% sucrose (Minoli et al., 2012). This suggests that previous gustatory experiments are not neutral for sucrose responsiveness.

### Neurophysiological Substrates

Gustatory receptors and the downward neurophysiological pathways have been reported on antennae, proboscis, and legs (Jørgensen et al., 2006, 2007; Calas et al., 2009; Kvello et al., 2010; Zhang et al., 2010; Popescu et al., 2013; Agnihotri et al., 2016; Seada et al., 2018); these neurophysiological results are consistent with our behavioral observations, as they indicated that sugar-induced neuronal responses are lower in the legs than in the proboscis, and glucose alone elicits less responses than sucrose or fructose. Quinine was also well detected but not by the neurons responding to sucrose (Jørgensen et al., 2007). This suggests its effect result from a central integration, rather than an interaction at the level of gustatory receptors; this is different in fruit flies, where bitter compounds can interact at the level of the receptor (Meunier et al., 2003; Jeong et al., 2013; French et al., 2015a,b). Popescu et al. (2013) observed sex-dependent responses, suggesting the differences we observed in Figure 1B (but not in Figure 6) are supported by differing response levels.

To fully understand how sugars are perceived and responded to by *A. ipsilon* and other moths, it will be necessary to describe the full neuronal pathway of information from antennal gustatory receptors to PER release. At the level of antennae, characterization of the receptors has already been started in moths and involves description of the antennal gustatory sensilla that house gustatory neurons as well as molecular description of the receptors and their interactions with sugars (Balkenius and Kelber, 2006; Popescu et al., 2013; Seada, 2015; Agnihotri et al., 2016; Seada et al., 2018); it would be particularly interesting to describe interactions in mixtures of sugars, quinine, and/or amino acid, specially to find out whether synergy or masking occurs, as for odors (Rospars, 2013); a tempting hypothesis is that glucose would act as a modulator of sugar receptor, explaining it elicits little response while favoring response to other sugars. In turn, gustatory information projects to the central nervous system, particularly the suboesophageal ganglion (Jørgensen et al., 2006; Kvello et al., 2006, 2010; Popescu et al., 2013). How motivation-linked information (hunger and/or other factors) is then integrated with gustatory input to elicit (or not) a PER remains to be determined. While electrophysiological and molecular tools will be needed to reach this goal, the sucrose responsiveness assay provides a solid framework to guide such research.

As previously discussed, stimulating legs with sucrose is much less prone to elicit a PER than stimulating antennae. Assuming motivation to release a PER and the corresponding motor control are central processes, a simple explanation is that legs bear less sugar receptors than antennae and/or that they bear different receptors (i.e., less sensitive to sucrose in the legs). Alternatively, gustatory pathways from the legs could be somehow less connected to the release of PER than those from antennae. Once again, electrophysiology (recording of gustatory sensilla from legs and antennae) will be helpful to distinguish between these two hypotheses, by determining whether gustatory responses of legs and antennae are comparable (Calas et al., 2009; Zhang et al., 2010).

Finally, understanding the central integration of sucrose input that leads to PER will be the ultimate goal. A way to approach this objective will be to understand which neurotransmitter systems are involved. Promising candidates are biogenic amines such as octopamine and dopamine, as they have well-known roles on sucrose sensitivity in other insects (Pankiw and Page, 2003; Schwaerzel et al., 2003; Unoki et al., 2005, 2006; Scheiner et al., 2006, 2014; Mizunami et al., 2009; Matsumoto et al., 2015). Interestingly, biogenic amines also modulate sex-pheromone-elicited behaviors in male moths (Linn and Roelofs, 1986; Linn et al., 1992; Pophof, 2000; Flecke and Stengl, 2009; Jarriault et al., 2009; Duportets et al., 2010; Abrieux et al., 2014; Hillier and Kavanagh, 2015), while sex pheromones do not affect sucrose responsiveness (Hostachy et al., submitted). If these neurotransmitters modulated sucrose responsiveness, this would imply that they do so through ways different from pheromone-responsiveness modulation.

## Data Availability Statement

All datasets generated for this study are included in the article/supplementary material.

## Author Contributions

CH, ND, and MD designed the experiments. CH and MD prepared the figures and analyzed the data. MD wrote the first draft of the manuscript. All authors performed the experiments and contributed to manuscript revision, read and approved the submitted version.

### Conflict of Interest

The authors declare that the research was conducted in the absence of any commercial or financial relationships that could be construed as a potential conflict of interest.

## Abbreviations

PER: Proboscis extension response.
