# Supplementary material for: Responsiveness to Sugar Solutions in the Moth Agrotis ipsilon: Parameters Affecting Proboscis Extension
Source: Front Physiol. 2019 Nov 26;10:1423. doi: 10.3389/fphys.2019.01423 (PMC6888557; doi:10.3389/fphys.2019.01423)
Supplement: Supplementary file 1 [file Data_Sheet_1.pdf]

SUPPLEMENTARY DATA

Supplementary Tables S1-12 report the details of statistics for Figure 1-6.

Supplementary Figures S1-6 report the same data as Figures 1-6, but with the animals spontaneously responding to water.

Supplementary Tables S13-24 report the details of statistics for Supplementary Figures S1-6.

		Sucrose concentration							
		**0%**	**0.1%**	**0.3%**	**1%**	**3%**	**10%**	**30%**	**0%**
**Global tests**	4 degrees of freedom χ^2				37.268	37.01	18.49	15.138	
	χ^2 p-value				< 0.001	< 0.001	< 0.001	0.004	
	Fisher's exact test p-value	1	0.149	0.126					0.163
**Pairwise comparisons (adjusted p-values)**		**0%**	**0.1%**	**0.3%**	**1%**	**3%**	**10%**	**30%**	**0%**
	5days/unfed 5days vs. 5days /unfed 3days				0.175	1	1	0.677	
	5days/unfed 5days vs. 5days /fed				< 0.001	0.124	0.184	0.614	
	5days/unfed 5days vs. 3days/unfed 3days				0.091	1	1	1	
	5days/unfed 5days vs. 3days /fed				< 0.001	< 0.001	0.054	0.677	
	5days /unfed 3days vs. 5days/fed				0.050	0.026	0.051	0.013	
	5days /unfed 3days vs. 3days/unfed 3days				0.880	1	1	0.677	
	5days /unfed 3days vs. 3days/fed				0.002	< 0.001	0.010	0.026	
	5days /fed vs. 3days/unfed 3days				0.166	0.011	0.174	0.614	
	5days /fed vs. 3days/fed				0.880	0.159	1	1	
	3days/unfed 3days vs. 3days /fed				0.015	< 0.001	0.051	0.677	

Supplementary Table S1. Detailed analysis of data from Figure 1A.

		Sucrose concentration							
		**0%**	**0.1%**	**0.3%**	**1%**	**3%**	**10%**	**30%**	**0%**
**Males vs. females (both 5days/unfed 5days)**	1 degree of freedom χ^2			6.358	3.283	0.010			0.213
	χ^2 p-value			0.012	0.070	.919			0.644
	Fisher's exact test p-value	1	0.022				1	1	
		**0%**	**0.1%**	**0.3%**	**1%**	**3%**	**10%**	**30%**	**0%**
**Global tests between females**	4 degrees of freedom χ^2				10.398	23.744	4.065		4.970
	χ^2 p-value				0.034	< 0.001	0.397		0.290
	Fisher's exact test p-value	1	0.231	0.046				0.020	
		**0%**	**0.1%**	**0.3%**	**1%**	**3%**	**10%**	**30%**	**0%**
**Pairwise comparisons between females (adjusted p-values)**	5days/unfed 5days vs. 5days /unfed 3days			1	1	0.036		1	
	5days/unfed 5days vs. 5days /fed			0.699	0.056	< 0.001		0.272	
	5days/unfed 5days vs. 3days/unfed 3days			1	1	0.258		1	
	5days/unfed 5days vs. 3days /fed			1	1	0.001		1	
	5days /unfed 3days vs. 5days/fed			0.483	0.121	0.275		1	
	5days /unfed 3days vs. 3days/unfed 3days			1	1	0.820		1	
	5days /unfed 3days vs. 3days/fed			0.981	1	0.755		1	
	5days /fed vs. 3days/unfed 3days			0.134	0.020	0.069		1	
	5days /fed vs. 3days/fed			1	0.331	0.820		0.272	
	3days/unfed 3days vs. 3days /fed			0.463	1	0.275		1	

Supplementary Table S2. Detailed analysis of data from Figure 1B.

		Sucrose concentration							
		**0%**	**0.1%**	**0.3%**	**1%**	**3%**	**10%**	**30%**	**0%**
**Global tests**	3 degrees of freedom χ^2		5.672	4.314	15.581	37.939			4.686
	χ^2 p-value		0.129	0.229	0.001	< 0.001			0.196
	Fisher's exact test p-value	1					< 0.001	0.201	
**Pairwise comparisons (adjusted p-values)**		**0%**	**0.1%**	**0.3%**	**1%**	**3%**	**10%**	**30%**	**0%**
	Unfed control vs. unfed random				0.37	0.71	1		
	Unfed control vs. fed control				0.282	< 0.001	0.094		
	Unfed control vs. fed random				0.282	< 0.001	0.078		
	Unfed random vs. fed control				0.012	< 0.001	0.030		
	Unfed random vs. fed random				0.006	< 0.001	0.010		
	Fed control vs. fed random				0.707	0.710	1		

Supplementary Table S3. Detailed analysis of data from Figure 2A.

		Sucrose concentration							
		**0%**	**0.1%**	**0.3%**	**1%**	**3%**	**10%**	**30%**	**0%**
	3 degrees of freedom χ^2					1.345	2.666	1.610	
**Global tests**	χ^2 p-value					0.718	0.446	0.657	
	Fisher's exact test p-value	1	0.064	0.068	0.417				.733

Supplementary Table S4. Detailed analysis of data from Figure 2B.

		Sucrose concentration							
		**0%**	**0.1%**	**0.3%**	**1%**	**3%**	**10%**	**30%**	**0%**
**Antennal stimulated vs. leg stimulated**	1 degree of freedom χ^2		8.011	10.634	20.267	23.945			12.267
	χ^2 p-value		0.005	0.001	< 0.001	< 0.001			< 0.001
	Fisher's exact test p-value	1					0.0002	0.001	
		**0%**	**0.1%**	**0.3%**	**1%**	**3%**	**10%**	**30%**	**0%**
**Antennal stimulation in both groups**	1 degree of freedom χ^2		1.609	1.267	0.003				0.069
	χ^2 p-value		0.205	0.260	0.955				0.792
	Fisher's exact test p-value	1				0.709	0.474	0.474	

Supplementary Table S5. Detailed analysis of data from Figure 3.

		Sucrose concentration							
		**0%**	**0.1%**	**0.3%**	**1%**	**3%**	**10%**	**30%**	**0%**
**Comparison before proboscis stimulation**	1 degree of freedom χ^2				2.375	0.472	0.075	0.472	
	χ^2 p-value				0.123	0.492	0.785	0.492	
	Fisher's exact test p-value	1	0.421	0.507					0.349
		**0%**	**0.1%**	**0.3%**	**1%**	**3%**	**10%**	**30%**	**0%**
**Comparison before and after water stimulation**	McNemar's test	0	0	0	0	0	0	0	0
	McNemar's test p-value	1	1	1	1	1	1	1	1
		**0%**	**0.1%**	**0.3%**	**1%**	**3%**	**10%**	**30%**	**0%**
**Comparison before and after quinine stimulation**	McNemar's test	0	0.333	0.667	3.571	3.267	9.783	8.000	1.000
	McNemar's test p-value	1	0.564	0.414	0.059	0.071	0.002	0.005	0.317

Supplementary Table S6. Detailed analysis of data from Figure 4A.

		Sucrose concentration							
		**0%**	**0.1%**	**0.3%**	**1%**	**3%**	**10%**	**30%**	**0%**
**Global test**	1 degree of freedom χ^2				21.582	63.828	83.977	68.646	
	χ^2 p-value				< 0.001	< 0.001	< 0.001	< 0.001	
	Fisher's exact test p-value	1.000	0.011	0.024					0.052

Supplementary Table S7. Detailed analysis of data from Figure 4B.

		Sugar concentration							
		**0%**	**0.1%**	**0.3%**	**1%**	**3%**	**10%**	**30%**	**0%**
**Global tests**	3 degrees of freedom χ^2			23.811	24.825	8.874	12.002		
	χ^2 p-value			< 0.001	< 0.001	0.012	0.002		
	Fisher's exact test p-value	1.000	0.002					0.003	0.430
**Pairwise comparisons (adjusted p-values)**		**0%**	**0.1%**	**0.3%**	**1%**	**3%**	**10%**	**30%**	**0%**
	Sucrose vs. Fructose		1.000	0.015	0.046	0.068	0.003	0.041	
	Sucrose vs. Sucrose/Fructose		0.030	0.015	0.003	0.463	0.217	1.000	
	Fructose vs. Sucrose/Fructose		0.016	< 0.001	< 0.001	0.015	0.217	0.014	

Supplementary Table S8. Detailed analysis of data from Figure 5A.

		Sugar concentration							
		**0%**	**0.1%**	**0.3%**	**1%**	**3%**	**10%**	**30%**	**0%**
**Global tests**	3 degrees of freedom χ^2		8.025	8.184	19.17	41.776	50.648		8.673
	χ^2 p-value		0.018	0.017	< 0.001	< 0.001	< 0.001		0.013
	Fisher's exact test p-value	1						< 0.001	
		**0%**	**0.1%**	**0.3%**	**1%**	**3%**	**10%**	**30%**	**0%**
**Pairwise comparisons (adjusted p-values)**	Sucrose vs. Glucose		0.017	0.020	< 0.001	< 0.001	< 0.001	< 0.001	0.022
	Sucrose vs. Sucrose/Glucose		0.956	0.974	0.813	0.805	0.686	1.000	0.737
	Glucose vs. Sucrose/Glucose		0.017	0.020	< 0.001	< 0.001	< 0.001	< 0.001	0.012

Supplementary Table S9. Detailed analysis of data from Figure 5B.

		Sugar concentration							
		**0%**	**0.1%**	**0.3%**	**1%**	**3%**	**10%**	**30%**	**0%**
**Global tests**	3 degrees of freedom χ^2					17.107	16.122	6.52	0.259
	χ^2 p-value					< 0.001	< 0.001	0.038	.879
	Fisher's exact test p-value	1	0.051	.588	.680				
**Pairwise comparisons (adjusted p-values)**		**0%**	**0.1%**	**0.3%**	**1%**	**3%**	**10%**	**30%**	**0%**
	Fructose vs. Glucose					< 0.001	0.001	0.335	
	Glucose vs. Fructose/Glucose					0.031	0.001	0.055	
	Fructose vs. Fructose/Glucose					0.049	0.980	0.490	

Supplementary Table S10. Detailed analysis of data from Figure 5C.

		Sugar concentration							
		**0%**	**0.1%**	**0.3%**	**1%**	**3%**	**10%**	**30%**	**0%**
**Global tests**	4 degrees of freedom χ^2			13.762	21.806	54.211	31.305		3.224
	χ^2 p-value			0.003	< 0.001	< 0.001	< 0.001		0.358
	Fisher's exact test p-value	1	0.578					< 0.001	
**Pairwise comparisons (adjusted p-values)**		**0%**	**0.1%**	**0.3%**	**1%**	**3%**	**10%**	**30%**	**0%**
	Sucrose vs. Glucose			0.072	< 0.001	< 0.001	< 0.001	< 0.001	
	Sucrose vs. Fructose			0.009	0.040	0.016	0.850	0.726	
	Sucrose vs. Mixture			0.598	0.307	0.151	1.000	0.726	
	Glucose vs. Fructose			0.600	0.068	< 0.001	< 0.001	0.045	
	Glucose vs. Mixture			0.490	0.002	< 0.001	< 0.001	0.045	
	Fructose vs. Mixture			0.235	0.221	0.291	1.000	1.000	

Supplementary Table S11. Detailed analysis of data from Figure 5D.

		Sugar concentration							
		**0%**	**0.1%**	**0.3%**	**1%**	**3%**	**10%**	**30%**	**0%**
**Males vs. females (sucrose)**	1 degree of freedom χ^2		0.033	0.091	0.063	0.034			0.012
	χ^2 p-value		.855	.763	.801	.853			.913
	Fisher's exact test p-value						0.060	0.060	
		**0%**	**0.1%**	**0.3%**	**1%**	**3%**	**10%**	**30%**	**0%**
**Global tests between females**	4 degrees of freedom χ^2			8.344	35.956	70.795	68.228		8.344
	χ^2 p-value			0.080	< 0.001	< 0.001	< 0.001		0.080
	Fisher's exact test p-value		0.524					0.004	
		**0%**	**0.1%**	**0.3%**	**1%**	**3%**	**10%**	**30%**	**0%**
**Pairwise comparisons between females (adjusted p-values)**	Sucrose vs. Fructose				0.818	1.000	1.000	0.420	
	Sucrose vs. Sucrose/Fructose				0.818	1.000	1.000	0.113	
	Sucrose vs. Sucrose/Fructose/Glucose				0.301	1.000	1.000	0.814	
	Sucrose vs. Glucose				< 0.001	< 0.001	< 0.001	1.000	
	Fructose vs. Sucrose/Fructose				0.197	1.000	1.000	1.000	
	Fructose vs. Sucrose/Fructose/Glucose				0.029	1.000	1.000	1.000	
	Fructose vs. Glucose				0.003	< 0.001	< 0.001	0.355	
	Sucrose/Fructose vs. Sucrose/Fructose/Glucose				0.818	1.000	1.000	0.951	
	Sucrose/Fructose vs. Glucose				< 0.001	< 0.001	< 0.001	0.064	
	Sucrose/Fructose/Glucose vs. Glucose				< 0.001	< 0.001	< 0.001	0.814	

Supplementary Table S12. Detailed analysis of data from Figure 6.

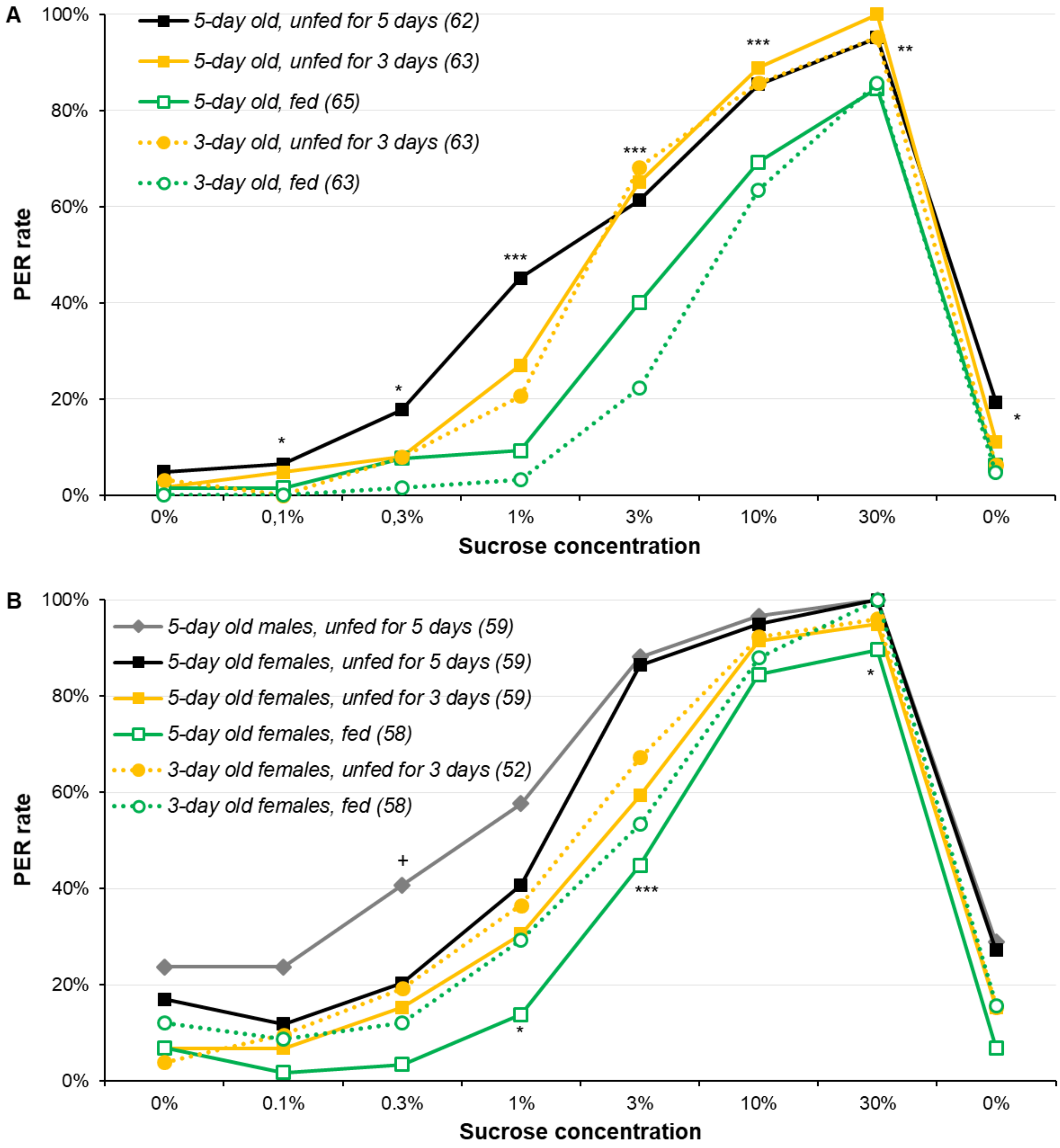

Supplementary Figure S1: data of Figure 1 including animals spontaneously responding to water. Details of the analyses are reported in Supplementary Tables S13 and S14. All other details as in Figure 1.

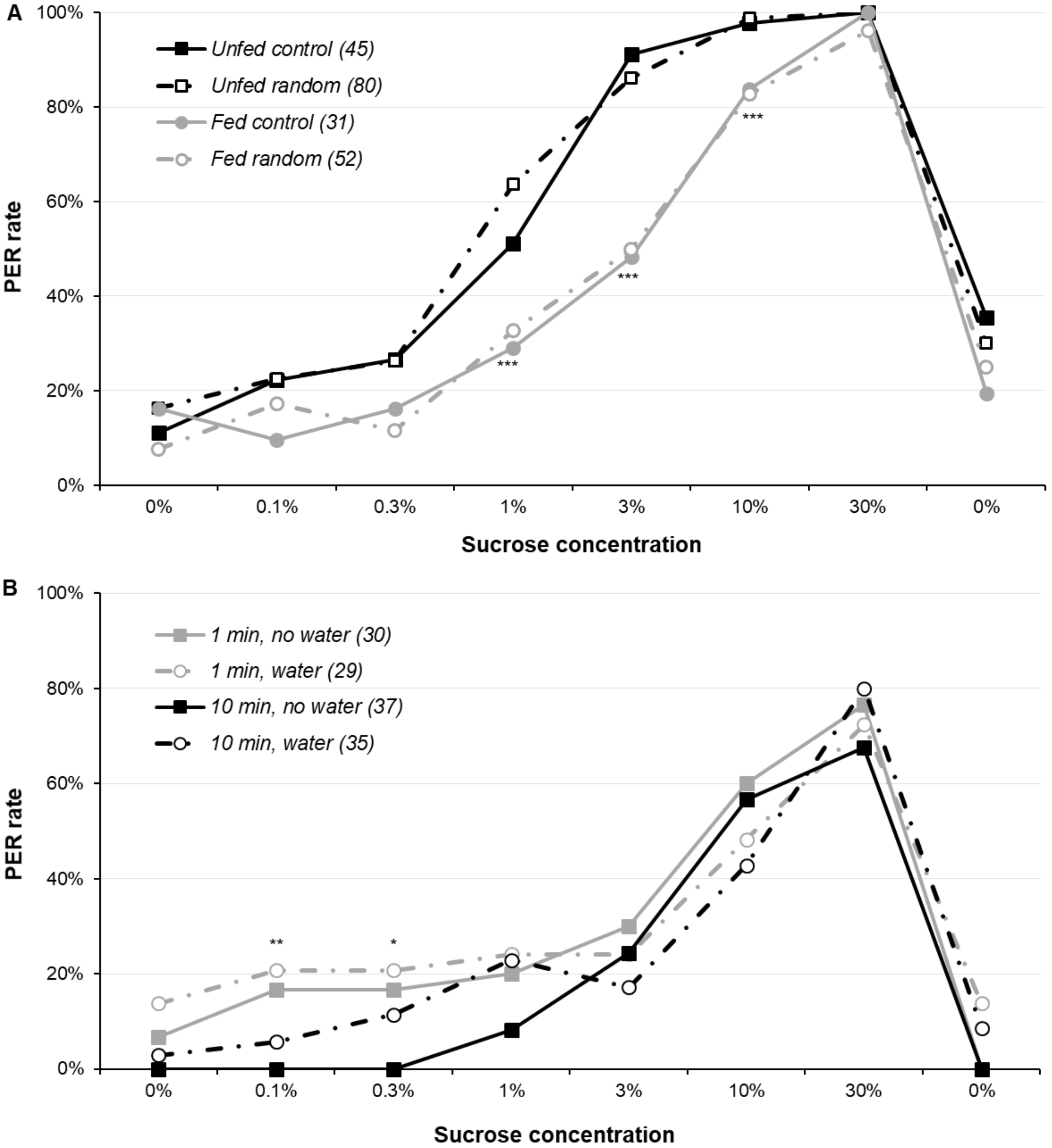

Supplementary Figure S2: data of Figure 2 including animals spontaneously responding to water. Details of the analyses are reported in Supplementary Tables S15 and S16. All other details as in Figure 2.

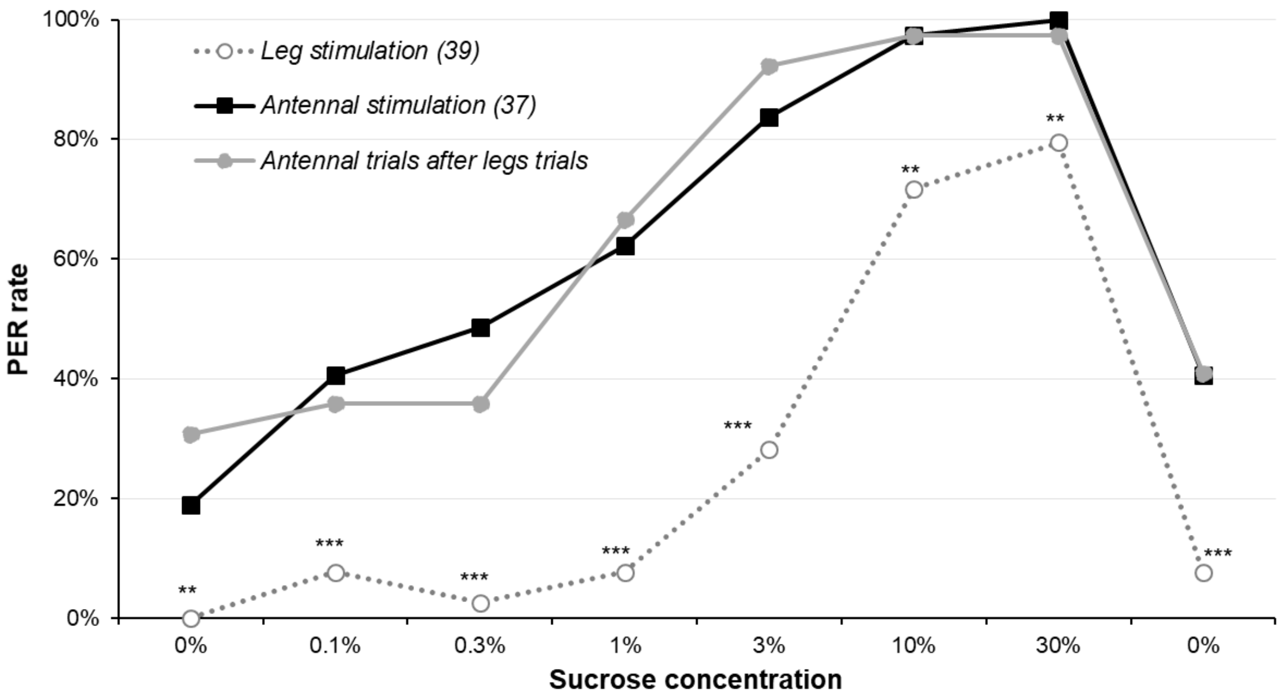

Supplementary Figure S3: data of Figure 3 including animals spontaneously responding to water. Details of the analyses are reported in Supplementary Table S17. All other details as in Figure 3.

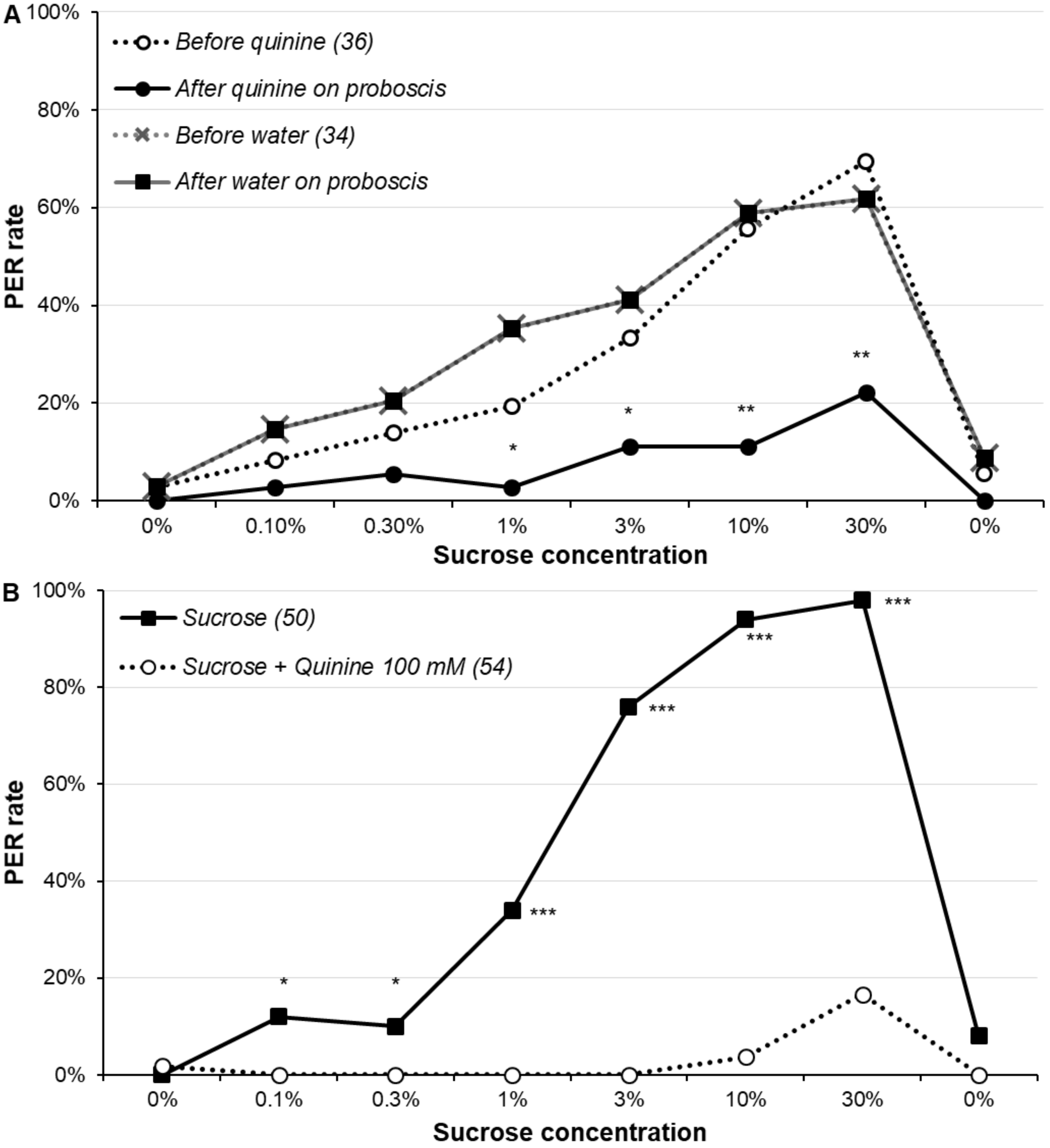

Supplementary Figure S4: data of Figure 4 including animals spontaneously responding to water. In part A, stars denote a significant difference between the PER rate before and after touching the proboscis with quinine, i.e. a significant rate of proboscis retraction (McNemar's test; *: $p < 0.050$, **: $p < 0.010$), and details of the analyses are reported in Supplementary Tables S18. In part B, stars denote a significant difference between the PER rate of the two groups (χ^2; *: $p < 0.050$, ***: $p < 0.001$), and details of the analyses are reported in Supplementary Tables S19. All other details as in Figure 4.

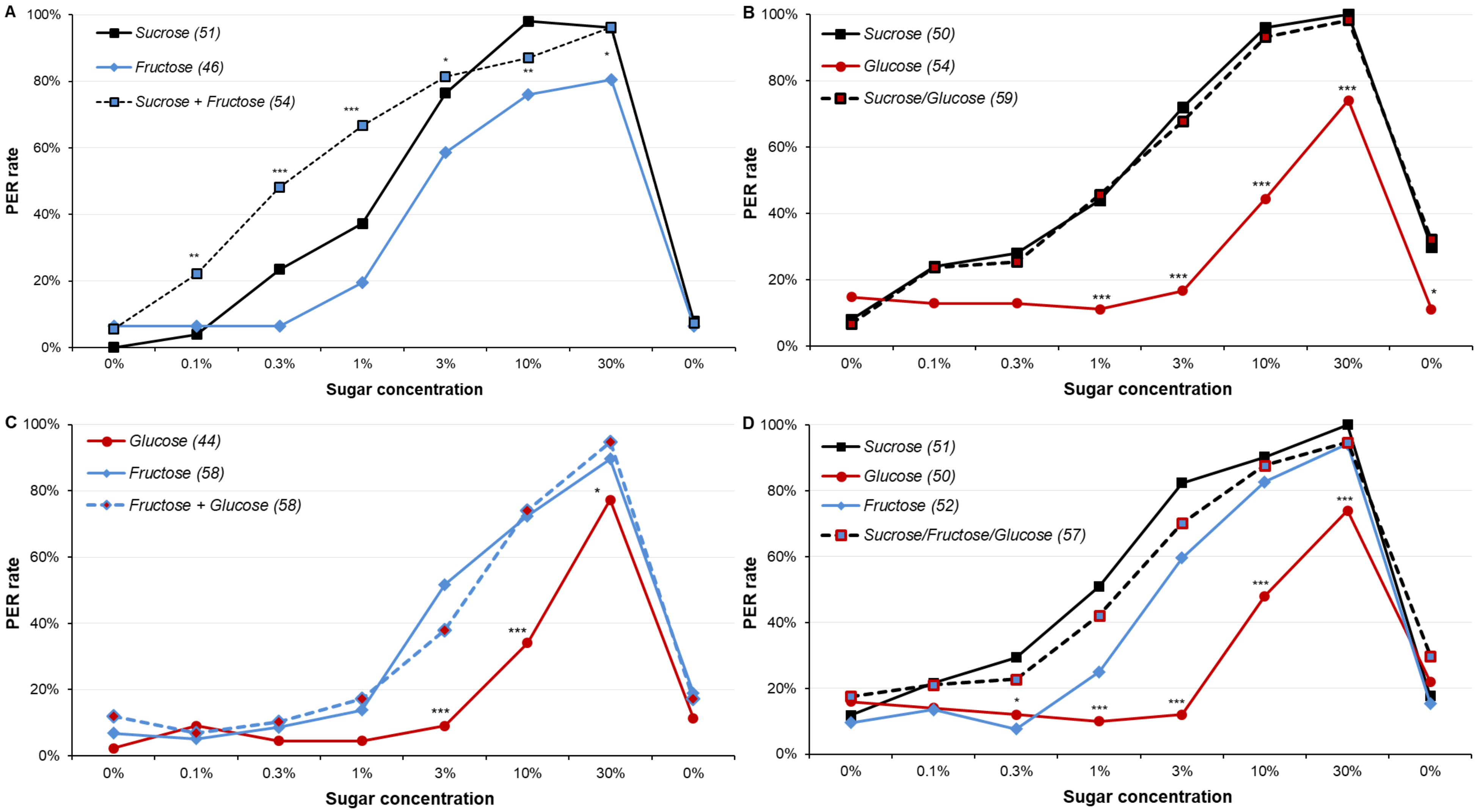

Supplementary Figure S5: data of Figure 5 including animals spontaneously responding to water. Details of the analyses are reported in Supplementary Tables S20 to S23. All other details as in Figure 5.

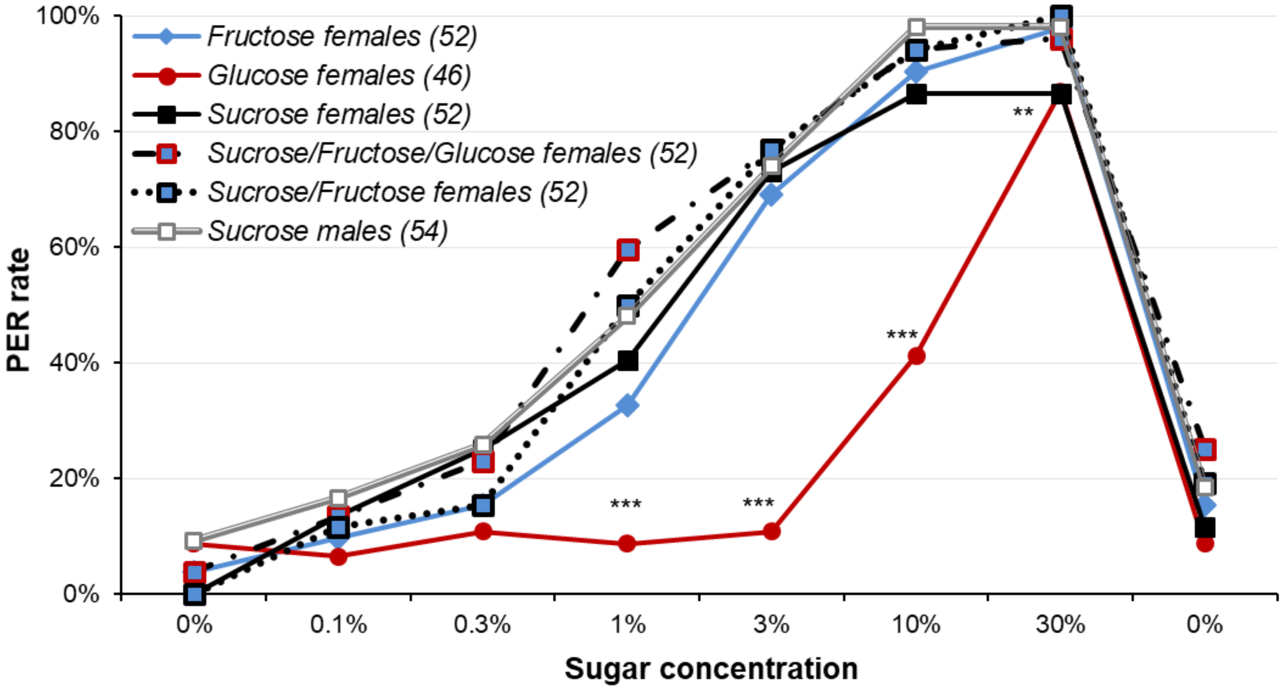

Supplementary Figure S6: data of Figure 6 including animals spontaneously responding to water. Details of the analyses are reported in Supplementary Tables S24. All other details as in Figure 6.

		Sucrose concentration							
		**0%**	**0.1%**	**0.3%**	**1%**	**3%**	**10%**	**30%**	**0%**
**Global tests**	4 degrees of freedom χ^2			10.734	40.835	39.155	19.467		10.419
	χ^2 p-value			0.030	< 0.001	< 0.001	< 0.001		0.034
	Fisher's exact test p-value	0.352	0.042					0.002	
**Pairwise comparisons (adjusted p-values)**		**0%**	**0.1%**	**0.3%**	**1%**	**3%**	**10%**	**30%**	**0%**
	5days/unfed 5days vs. 5days /unfed 3days	1	1	0.165	1	1	0.714	1	1
	5days/unfed 5days vs. 5days /fed	1	1	< 0.001	0.082	0.156	0.611	0.291	1
	5days/unfed 5days vs. 3days/unfed 3days	0.576	1	0.030	1	1	1	0.291	0.576
	5days/unfed 5days vs. 3days /fed	0.576	0.021	< 0.001	< 0.001	0.039	0.714	0.139	0.576
	5days /unfed 3days vs. 5days/fed	1	1	0.056	0.027	0.045	0.013	1	1
	5days /unfed 3days vs. 3days/unfed 3days	1	1	0.548	1	1	0.732	1	1
	5days /unfed 3days vs. 3days/fed	1	1	0.002	< 0.001	0.008	0.026	1	1
	5days /fed vs. 3days/unfed 3days	1	1	0.254	0.009	0.156	0.611	1	1
	5days /fed vs. 3days/fed	1	1	0.548	0.120	1	1	1	1
	3days/unfed 3days vs. 3days /fed	1	1	0.030	< 0.001	0.037	0.714	1	1

Supplementary Table S13. Detailed analysis of data from Supplementary Figure S1A.

		Sucrose concentration							
		**0%**	**0.1%**	**0.3%**	**1%**	**3%**	**10%**	**30%**	**0%**
**Males vs. females (both 5days/unfed 5days)**	1 degree of freedom χ^2	0.837	2.839	5.756	3.391	0.076			0.042
	χ^2 p-value	.360	0.092	0.016	0.066	.782			.838
	Fisher's exact test p-value						1	1	
		**0%**	**0.1%**	**0.3%**	**1%**	**3%**	**10%**	**30%**	**0%**
**Global tests between females**	4 degrees of freedom χ^2	7.191		8.778	11.521	24.874	4.349		9.013
	χ^2 p-value	.126		0.067	0.021	< 0.001	.361		0.061
	Fisher's exact test p-value		.256					0.012	
		**0%**	**0.1%**	**0.3%**	**1%**	**3%**	**10%**	**30%**	**0%**
**Pairwise comparisons between females (adjusted p-values)**	5days/unfed 5days vs. 5days /unfed 3days				1	0.007		1	
	5days/unfed 5days vs. 5days /fed				0.011	< 0.001		0.129	
	5days/unfed 5days vs. 3days/unfed 3days				1	0.112		1	
	5days/unfed 5days vs. 3days /fed				1	< 0.001		1	
	5days /unfed 3days vs. 5days/fed				0.237	0.583		1	
	5days /unfed 3days vs. 3days/unfed 3days				1	1		1	
	5days /unfed 3days vs. 3days/fed				1	1		1	
	5days /fed vs. 3days/unfed 3days				0.051	0.112		1	
	5days /fed vs. 3days/fed				0.295	1		0.246	
	3days/unfed 3days vs. 3days /fed				1	0.583		1	

Supplementary Table S14. Detailed analysis of data from Supplementary Figure S1B

		Sucrose concentration							
		**0%**	**0.1%**	**0.3%**	**1%**	**3%**	**10%**	**30%**	**0%**
**Global tests**	3 degrees of freedom χ^2	2.456	2.755	5.417	17.47	37.729			2.779
	χ^2 p-value	0.483	0.431	0.144	< 0.001	< 0.001			0.427
	Fisher's exact test p-value						< 0.001	0.129	
**Pairwise comparisons (adjusted p-values)**		**0%**	**0.1%**	**0.3%**	**1%**	**3%**	**10%**	**30%**	**0%**
	Unfed control vs. unfed random				0.335	0.844	1		
	Unfed control vs. fed control				0.222	< 0.001	0.115		
	Unfed control vs. fed random				0.222	< 0.001	0.073		
	Unfed random vs. fed control				0.005	< 0.001	0.032		
	Unfed random vs. fed random				0.003	< 0.001	0.006		
	Fed control vs. fed random				0.728	0.887	1		

Supplementary Table S15. Detailed analysis of data from Supplementary Figure S2A.

		Sucrose concentration							
		**0%**	**0.1%**	**0.3%**	**1%**	**3%**	**10%**	**30%**	**0%**
**Global tests**	3 degrees of freedom χ^2				3.773	1.503	2.437	1.601	
	χ^2 p-value				0.287	0.682	0.487	0.659	
	Fisher's exact test p-value	0.055	0.008	0.016					0.209
**Pairwise comparisons (adjusted p-values)**		**0%**	**0.1%**	**0.3%**	**1%**	**3%**	**10%**	**30%**	**0%**
	1 min, no water vs. 1 min, water		0.748	1.000					
	1 min, no water vs. 10 min, no water		0.074	0.074					
	1 min, no water vs. 10 min, water		0.698	1.000					
	1 min, water vs. 10 min, no water		0.031	0.031					
	1 min, water vs. 10 min, water		0.506	1.000					
	10 min, no water vs. 10 min, water		0.698	0.204					

Supplementary Table S16. Detailed analysis of data from Supplementary Figure S2B.

		Sucrose concentration							
		**0%**	**0.1%**	**0.3%**	**1%**	**3%**	**10%**	**30%**	**0%**
**Antennal stimulated vs. leg stimulated**	1 degree of freedom χ^2		11.335	21.506	25.029	23.720	9.287		11.335
	χ^2 p-value		< 0.001	< 0.001	< 0.001	< 0.001	0.002		< 0.001
	Fisher's exact test p-value	0.005						0.005	
		**0%**	**0.1%**	**0.3%**	**1%**	**3%**	**10%**	**30%**	**0%**
**Antennal stimulation in both groups**	1 degree of freedom χ^2	1.422	0.173	1.266	0.168				0.002
	χ^2 p-value	0.233	0.677	0.260	0.681				0.966
	Fisher's exact test p-value					.304	1	1	

Supplementary Table S17. Detailed analysis of data from Supplementary Figure S3.

		Sucrose concentration							
		**0%**	**0.1%**	**0.3%**	**1%**	**3%**	**10%**	**30%**	**0%**
**Comparison before proboscis stimulation**	1 degree of freedom χ^2			0.553	2.221	0.461	0.076	0.458	
	χ^2 p-value			0.457	0.136	0.497	0.782	0.499	
	Fisher's exact test p-value	1	0.4717						0.669
		**0%**	**0.1%**	**0.3%**	**1%**	**3%**	**10%**	**30%**	**0%**
**Comparison before and after water stimulation**	McNemar's test	0	0	0	0	0	0	0	0
	McNemar's test p-value	1	1	1	1	1	1	1	1
		**0%**	**0.1%**	**0.3%**	**1%**	**3%**	**10%**	**30%**	**0%**
**Comparison before and after quinine stimulation**	McNemar's test	1.000	1.000	1.286	4.500	4.000	10.667	8.758	2.000
	McNemar's test p-value	0.317	0.317	0.257	0.034	0.046	0.0011	0.003	0.157

Supplementary Table S18. Detailed analysis of data from Supplementary Figure S4A.

		Sucrose concentration							
		**0%**	**0.1%**	**0.3%**	**1%**	**3%**	**10%**	**30%**	**0%**
**Global test**	1 degree of freedom χ^2				21.948	64.669	84.953	69.622	
	χ^2 p-value				< 0.001	< 0.001	< 0.001	< 0.001	
	Fisher's exact test p-value	1	0.010	0.023					0.050

Supplementary Table S19. Detailed analysis of data from Supplementary Figure S4B.

		Sugar concentration							
		**0%**	**0.1%**	**0.3%**	**1%**	**3%**	**10%**	**30%**	**0%**
**Global tests**	3 degrees of freedom χ^2		10.278	22.271	23.397	7.031	10.607		
	χ^2 p-value		0.006	< 0.001	< 0.001	0.030	0.005		
	Fisher's exact test p-value	0.189						0.012	1.000
		**0%**	**0.1%**	**0.3%**	**1%**	**3%**	**10%**	**30%**	**0%**
**Pairwise comparisons (adjusted p-values)**	Sucrose vs. Fructose		0.666	0.021	0.055	0.122	0.004	0.064	
	Sucrose vs. Sucrose/Fructose		0.025	0.017	0.005	0.528	0.122	1.000	
	Fructose vs. Sucrose/Fructose		0.094	< 0.001	< 0.001	0.037	0.195	0.064	

Supplementary Table S20. Detailed analysis of data from Supplementary Figure S5A.

		Sugar concentration							
		**0%**	**0.1%**	**0.3%**	**1%**	**3%**	**10%**	**30%**	**0%**
**Global tests**	3 degrees of freedom χ^2	2.325	2.654	4.010	18.537	40.929	52.69		7.935
	χ^2 p-value	.312	.265	.135	< 0.001	< 0.001	< 0.001		0.019
	Fisher's exact test p-value							< 0.001	
**Pairwise comparisons (adjusted p-values)**		**0%**	**0.1%**	**0.3%**	**1%**	**3%**	**10%**	**30%**	**0%**
	Sucrose vs. Glucose				< 0.001	< 0.001	< 0.001	< 0.001	0.033
	Sucrose vs. Sucrose/Glucose				0.854	.634	.685	1	0.805
	Glucose vs. Sucrose/Glucose				< 0.001	< 0.001	< 0.001	< 0.001	0.021

Supplementary Table S21. Detailed analysis of data from Supplementary Figure S5B.

		Sugar concentration							
		**0%**	**0.1%**	**0.3%**	**1%**	**3%**	**10%**	**30%**	**0%**
**Global tests**	3 degrees of freedom χ^2				3.826	20.333	20.935	7.571	1.128
	χ^2 p-value				.148	< 0.001	< 0.001	0.023	.569
	Fisher's exact test p-value	.178	.684	.668					
**Pairwise comparisons (adjusted p-values)**		**0%**	**0.1%**	**0.3%**	**1%**	**3%**	**10%**	**30%**	**0%**
	Fructose vs. Glucose					< 0.001	< 0.001	0.210	
	Glucose vs. Fructose/Glucose					0.002	< 0.001	0.043	
	Fructose vs. Fructose/Glucose					0.135	0.834	0.490	

Supplementary Table S22. Detailed analysis of data from Supplementary Figure S5C.

		Sugar concentration							
		**0%**	**0.1%**	**0.3%**	**1%**	**3%**	**10%**	**30%**	**0%**
**Global tests**	4 degrees of freedom χ^2	1.817	2.082	10.311	23.251	58.748	34.012		3.958
	χ^2 p-value	0.611	0.556	0.016	< 0.001	< 0.001	< 0.001		0.266
	Fisher's exact test p-value							< 0.001	
**Pairwise comparisons (adjusted p-values)**		**0%**	**0.1%**	**0.3%**	**1%**	**3%**	**10%**	**30%**	**0%**
	Sucrose vs. Glucose			0.194	< 0.001	< 0.001	< 0.001	< 0.001	
	Sucrose vs. Fructose			0.031	0.030	0.033	0.800	0.728	
	Sucrose vs. Mixture			1.000	0.360	0.279	0.918	0.728	
	Glucose vs. Fructose			1.000	0.141	< 0.001	< 0.001	0.026	
	Glucose vs. Mixture			0.616	0.001	< 0.001	< 0.001	0.026	
	Fructose vs. Mixture			0.180	0.141	0.279	0.918	1.000	

Supplementary Table S23. Detailed analysis of data from Supplementary Figure S5D.

		**Sugar concentration**							
		**0%**	**0.1%**	**0.3%**	**1%**	**3%**	**10%**	**30%**	**0%**
**Males vs. females (sucrose)**	1 degree of freedom χ^2		0.212	0.012	0.647	0.014			1.007
	χ^2 p-value		.645	.913	.421	.907			.316
	Fisher's exact test p-value	0.057					0.030	0.030	
		**0%**	**0.1%**	**0.3%**	**1%**	**3%**	**10%**	**30%**	**0%**
**Global tests between females**	4 degrees of freedom χ^2		1.7	4.676	30.61	65.104	66.16		6.101
	χ^2 p-value		.791	.322	< 0.001	< 0.001	< 0.001		0.192
	Fisher's exact test p-value	0.060						0.005	
		**0%**	**0.1%**	**0.3%**	**1%**	**3%**	**10%**	**30%**	**0%**
**Pairwise comparisons between females (adjusted p-values)**	Sucrose vs. Fructose				0.974	1.000	1.000	0.419	
	Sucrose vs. Sucrose/Fructose				0.974	1.000	1.000	0.113	
	Sucrose vs. Sucrose/Fructose/Glucose				0.249	1.000	1.000	0.853	
	Sucrose vs. Glucose				0.003	< 0.001	< 0.001	1.000	
	Fructose vs. Sucrose/Fructose				0.292	1.000	1.000	1.000	
	Fructose vs. Sucrose/Fructose/Glucose				0.035	1.000	1.000	1.000	
	Fructose vs. Glucose				0.027	< 0.001	< 0.001	0.390	
	Sucrose/Fructose vs. Sucrose/Fructose/Glucose				0.974	1.000	1.000	1.000	
	Sucrose/Fructose vs. Glucose				< 0.001	< 0.001	< 0.001	0.089	
	Sucrose/Fructose/Glucose vs. Glucose				0.000	0.000	0.000	0.853	

Supplementary Table S24. Detailed analysis of data from Supplementary Figure S6.
